# Global monitoring of antimicrobial resistance based on metagenomics analyses of urban sewage

**DOI:** 10.1038/s41467-019-08853-3

**Published:** 2019-03-08

**Authors:** Rene S. Hendriksen, Patrick Munk, Patrick Njage, Bram van Bunnik, Luke McNally, Oksana Lukjancenko, Timo Röder, David Nieuwenhuijse, Susanne Karlsmose Pedersen, Jette Kjeldgaard, Rolf S. Kaas, Philip Thomas Lanken Conradsen Clausen, Josef Korbinian Vogt, Pimlapas Leekitcharoenphon, Milou G. M. van de Schans, Tina Zuidema, Ana Maria de Roda Husman, Simon Rasmussen, Bent Petersen, The Global Sewage Surveillance project consortium, Artan Bego, Catherine Rees, Susan Cassar, Kris Coventry, Peter Collignon, Franz Allerberger, Teddie O. Rahube, Guilherme Oliveira, Ivan Ivanov, Yith Vuthy, Thet Sopheak, Christopher K. Yost, Changwen Ke, Huanying Zheng, Li Baisheng, Xiaoyang Jiao, Pilar Donado-Godoy, Kalpy Julien Coulibaly, Matijana Jergović, Jasna Hrenovic, Renáta Karpíšková, Jose Eduardo Villacis, Mengistu Legesse, Tadesse Eguale, Annamari Heikinheimo, Lile Malania, Andreas Nitsche, Annika Brinkmann, Courage Kosi Setsoafia Saba, Bela Kocsis, Norbert Solymosi, Thorunn R. Thorsteinsdottir, Abdulla Mohamed Hatha, Masoud Alebouyeh, Dearbhaile Morris, Martin Cormican, Louise O’Connor, Jacob Moran-Gilad, Patricia Alba, Antonio Battisti, Zeinegul Shakenova, Ciira Kiiyukia, Eric Ng’eno, Lul Raka, Jeļena Avsejenko, Aivars Bērziņš, Vadims Bartkevics, Christian Penny, Heraa Rajandas, Sivachandran Parimannan, Malcolm Vella Haber, Pushkar Pal, Gert-Jan Jeunen, Neil Gemmell, Kayode Fashae, Rune Holmstad, Rumina Hasan, Sadia Shakoor, Maria Luz Zamudio Rojas, Dariusz Wasyl, Golubinka Bosevska, Mihail Kochubovski, Cojocaru Radu, Amy Gassama, Vladimir Radosavljevic, Stefan Wuertz, Rogelio Zuniga-Montanez, Moon Y. F. Tay, Dagmar Gavačová, Katarina Pastuchova, Peter Truska, Marija Trkov, Kerneels Esterhuyse, Karen Keddy, Marta Cerdà-Cuéllar, Sujatha Pathirage, Leif Norrgren, Stefan Örn, D. G. Joakim Larsson, Tanja Van der Heijden, Happiness Houka Kumburu, Bakary Sanneh, Pawou Bidjada, Berthe-Marie Njanpop-Lafourcade, Somtinda Christelle Nikiema-Pessinaba, Belkis Levent, John Scott Meschke, Nicola Koren Beck, Chinh Dang Van, Nguyen Do Phuc, Doan Minh Nguyen Tran, Geoffrey Kwenda, Djim-adjim Tabo, Astrid Louise Wester, Sara Cuadros-Orellana, Clara Amid, Guy Cochrane, Thomas Sicheritz-Ponten, Heike Schmitt, Jorge Raul Matheu Alvarez, Awa Aidara-Kane, Sünje J. Pamp, Ole Lund, Tine Hald, Mark Woolhouse, Marion P. Koopmans, Håkan Vigre, Thomas Nordahl Petersen, Frank M. Aarestrup

**Affiliations:** 1 National Food Institute, Technical University of Denmark, Kgs. Lyngby, 2800 Denmark; 2 Usher Institute, University of Edinburgh, Edinburgh, EH8 9AG UK; 3 Centre for Synthetic and Systems Biology, School of Biological Sciences, University of Edinburgh, Edinburgh, EH9 3JD UK; 4 Viroscience, Erasmus Medical Center, Rotterdam, 3015 The Netherlands; 5 RIKILT Wageningen University and Research, Wageningen, 6708 The Netherlands; 6 National Institute for Public Health and the Environment (RIVM), Bilthoven, 3721 The Netherlands; 7 Department of Bio and Health Informatics, Technical University of Denmark, Kgs. Lyngby, 2800 Denmark; 8 European Molecular Biology Laboratory, European Bioinformatics Institute, Hinxton, CB10 1SD UK; 9 Centre of Excellence for Omics-Driven Computational Biodiscovery, AIMST University, Kedah, 08100 Malaysia; 10 World Health Organization, Geneva, 1202 Switzerland; 11 Institute of Public Health, Tirana, 1000 Albania; 12 Melbourne Water Corporation, Melbourne, 3008 VIC Australia; 13 Canberra Hospital, Canberra, 2605 ACT Australia; 14 Austrian Agency for Health and Food Safety (AGES), Vienna, 1220 Austria; 15 Botswana International University of Science and Technology, Private Bag 16, Papapye, Botswana; 16 Vale Institute of Technology, Sustainable Development, Belém, 66055-090 Brazil; 17 National Center of Infectious and Parasitic Diseases, Sofia, 1504 Bulgaria; 18 Institut Pasteur du Cambodge, Phnom Penh, 855 Cambodia; 19 University of Regina, Regina, S4S0A2 Canada; 20 Guangdong Provincial Center for Disease Control and Prevention, No. 160, Guangzhou, 511430 China; 21 Shantou University Medical College, Shantou, 515041 China; 22 Corporacion Colombiana de Investigacion Agropecuaria (AGROSAVIA), Mosquera, 250040 Colombia; 23 Institut Pasteur de Côte d’Ivoire, Abidjan, 01 BP 490 Abidjan 01 Côte d’Ivoire; 24 Andrija Stampar Teaching Institute of Public Health, Zagreb, 10000 Croatia; 25 University of Zagreb, Zagreb, 10000 Croatia; 26 Veterinary Research Institute, Brno, 621 00 Czech Republic; 27 Instituto Nacional de Investigación en Salud Pública-INSPI (CRNRAM), Quito, 170136 Ecuador; 28 Addis Ababa University, Addis Ababa, P.O. Box 1176 Ethiopia; 29 University of Helsinki, Helsinki, FI00014 Finland; 30 National Center for Disease Control and Public Health, Tbilisi, 177 Georgia; 31 Robert Koch Institute, Berlin, 13353 Germany; 32 University for Development Studies, Tamale, 233 Ghana; 33 Semmelweis University, Institute of Medical Microbiology, Budapest, 1089 Hungary; 34 University of Veterinary Medicine, Budapest, 1078 Hungary; 35 Institute for Experimental Pathology, University of Iceland, Keldur, 112 Iceland; 36 Cochin University of Science and Technology, Cochin, 682016 India; 37 Foodborne and Waterborne Diseases Research center, Research Institute for Gastroenterology and Liver Diseases, Shahid Beheshti University of Medical Sciences, Tehran, 1985717413 Iran; 38 National University of Ireland Galway, Galway, H91 TK33 Ireland; 39 School of Public Health, Ben Gurion University of the Negev and Ministry of Health, Beer Sheva, 8410501 Israel; 40 Istituto Zooprofilattico Sperimentale del Lazio e della Toscana, Rome, 178 Italy; 41 National Center of Expertise, Taldykorgan, 40000 Kazakhstan; 42 Mount Kenya University, Thika, P. O. Box 342-01000 Kenya; 43 Kenya Medical Research Institute, Nairobi, 254 Kenya; 44 University of Prishtina “Hasan Prishtina” & National Institute of Public Health of Kosovo, Prishtina, 10000 Kosovo; 45 Institute of Food Safety, Riga, LV-1076 Latvia; 46 Luxembourg Institute of Science and Technology, Belvaux, L-4422 Luxembourg; 47 Environmental Health Directorate, St. Venera, SVR 9018 Malta; 48 Agriculture and Forestry University, Kathmandu, 44200 Nepal; 49 University of Otago, Dunedin, 9016 New Zealand; 50 University of Ibadan, Ibadan, 200284 Nigeria; 51 VEAS, Slemmestad, NO-3470 Norway; 52 Aga Khan University, Karachi, 74800 Pakistan; 53 National Institute of Health, Lima, 15072 Peru; 54 National Veterinary Research Institute, Pulawy, 24-100 Poland; 55 Institute of Public Health of the Republic of Macedonia, Skopje, 1000 Republic of Macedonia; 56 State Medical and Pharmaceutical University, Chişinău, MD-2004 Republic of Moldova; 57 Institut Pasteur de Dakar, Dakar, BP 220 Senegal; 58 Institute of Veterinary Medicine of Serbia, Belgrade, 11000 Serbia; 59 Nanyang Technological University, Singapore, Singapore Centre for Environmental Life Sciences Engineering (SCELSE), Singapore, 637551 Singapore; 60 Nanyang Technological University Food Technology Centre (NAFTEC), Nanyang Technological University (NTU), Singapore, 637551 Singapore; 61 Public Health Authority of the Slovak Republic, Bratislava, 826 45 Slovakia; 62 National Laboratory of Health, Environment and Food, Ljubjana, 1000 Slovenia; 63 Daspoort Waste Water Treatment Works, Pretoria, 1 South Africa; 64 University of the Witwatersrand, Johannesburg, 1485 South Africa; 65 IRTA, Centre de Recerca en Sanitat Animal (CReSA, IRTA-UAB), Bellaterra, 8193 Spain; 66 Medical Research Institute, Colombo, 800 Sri Lanka; 67 Swedish University of Agricultural Sciences, Uppsala, 75007 Sweden; 68 The Sahlgrenska Academy at the University of Gothenburg, Gothenburg, SE-413 46 Sweden; 69 Ara Region Bern Ag, Herrenschwanden, 3037 Switzerland; 70 Kilimanjaro Clinical Research Institute, Moshi, 255 Tanzania; 71 National Public Health Laboratories, Ministry of Health and Social Welfare, Kotu Layout, Kotu Layout, Kotu, 1863 The Gambia; 72 National Institute of Hygiene, Lome, 228 Togo; 73 Agence de Médecine Préventive, Dapaong, Togo; 74 Division of Integrated Surveillance of Health Emergencies and Response, Lome, Togo; 75 Public Health Institution of Turkey, Ankara, 6100 Turkey; 76 University of Washington, Seattle, 98105-6099 WA USA; 77 Institute of Public Health in Ho Chi Minh City, Ho Chi Minh City, 700000 Vietnam; 78 University of Zambia, Lusaka, 15101 Zambia; 79 University of N’Djamena, N’Djamena, 1117 Chad; 80 Norwegian Institute of Public Health, Oslo, 0456 Norway; 81 Universidad Católica del Maule, Av. San Miguel 3605, Talca, Chile; 82 Centro de Biotecnología de los Recursos Naturales, Facultad de Ciencias Agrárias y Forestales, Universidad Católica del Maule, Av. San Miguel 3605, Talca, Chile

## Abstract

Antimicrobial resistance (AMR) is a serious threat to global public health, but obtaining representative data on AMR for healthy human populations is difficult. Here, we use metagenomic analysis of untreated sewage to characterize the bacterial resistome from 79 sites in 60 countries. We find systematic differences in abundance and diversity of AMR genes between Europe/North-America/Oceania and Africa/Asia/South-America. Antimicrobial use data and bacterial taxonomy only explains a minor part of the AMR variation that we observe. We find no evidence for cross-selection between antimicrobial classes, or for effect of air travel between sites. However, AMR gene abundance strongly correlates with socio-economic, health and environmental factors, which we use to predict AMR gene abundances in all countries in the world. Our findings suggest that global AMR gene diversity and abundance vary by region, and that improving sanitation and health could potentially limit the global burden of AMR. We propose metagenomic analysis of sewage as an ethically acceptable and economically feasible approach for continuous global surveillance and prediction of AMR.

## Abstract (web summary)

Obtaining data on antimicrobial resistance (AMR) from healthy human populations is difficult. Here, Hendriksen et al. use metagenomic analysis to obtain AMR data from untreated sewage from 79 sites in 60 countries, finding correlations with socio-economic, health and environmental factors.

## Introduction

Antimicrobial resistance (AMR) is a cross-cutting and increasing threat to global health^1,2^, and it threatens to undermine decades of progress in the treatment of infectious diseases. AMR is a complex problem with multiple and interconnected drivers, which may include changing dynamics in travel, trade, climate change, and populations. Reliable information that accurately describes and characterizes the global occurrence and transmission of AMR is essential to address this challenge and to support national and global priority setting, public health actions, and treatment decisions.

Current surveillance of AMR is often focusing on a few pathogens only and mainly based on passive reporting of phenotypic laboratory results for specific pathogens isolated from human clinical infections^1,3–5^. This procedure leads to significant time delays, often incomparable data, and a narrow pathogen spectrum not capturing all relevant AMR genes, where the major part might be present in the commensal bacterial flora of healthy individuals. However, obtaining fecal samples from healthy humans is logistically difficult.

From a surveillance point of view, urban sewage is attractive because it provides sampling material from a large and mostly healthy population, which otherwise would not be feasible to monitor. Globally, a rapidly increased proportion of the human population live in urban areas^6^ and an increasing proportion is connected to a sewer system^7,8^. In addition, analyzing sewage samples does not require informed consent, thus limiting ethical concerns and has limited practical and logistical barriers for sampling. Most microbiological studies on sewage have focused on the risk of discharge of insufficiently treated sewage or problems related to heavy rainfall overflow, but some evaluations on the surveillance of pathogens have also been performed^9,10^. Additionally, sewage has proven useful for surveillance in the global polio eradication program^11,12^.

Metagenomic techniques, using short-read next-generation sequencing data, benefit from the ability to quantify thousands of especially transmissible resistance genes in a single sample. Moreover, it can provide additional information about the presence of bacterial species, pathogens, and virulence genes and the data can be reanalyzed if novel genes of interest are identified. It should, however, also be acknowledged that short-read metagenomics might provide limited information regarding the host of the genes or the genetic environment. Metagenomics has been found to be superior to conventional methods for AMR surveillance in pig herds^13^ and has also been utilized for the surveillance of global AMR gene dissemination through international flights^14^. Additionally, an extensively shared resistome was observed across urban sewage samples within China^15^, as well as between individuals and environmental samples in Lima, Peru^16^. Interestingly, Pehrsson et al.^16^ showed that even though changes in the bacterial composition were observed between feces and sewage, this was not the case for AMR genes.

Here we use metagenomic analysis of untreated sewage to characterize the bacterial resistome from 79 sites in 60 countries. We find systematic differences in abundance and diversity of AMR genes between Europe/North-America/Oceania and Africa/Asia/South-America. Antimicrobial use data and bacterial taxonomy only explain a minor part of the AMR variation that we observe. However, AMR gene abundance strongly correlates with socio-economic, health, and environmental factors, which we use to predict AMR gene abundances in all countries in the world. Our findings suggest that global AMR gene diversity and abundance vary by region and that improving sanitation and health could potentially limit the global burden of AMR.

## Results

### Global distribution of AMR genes

**Fig. 1:**
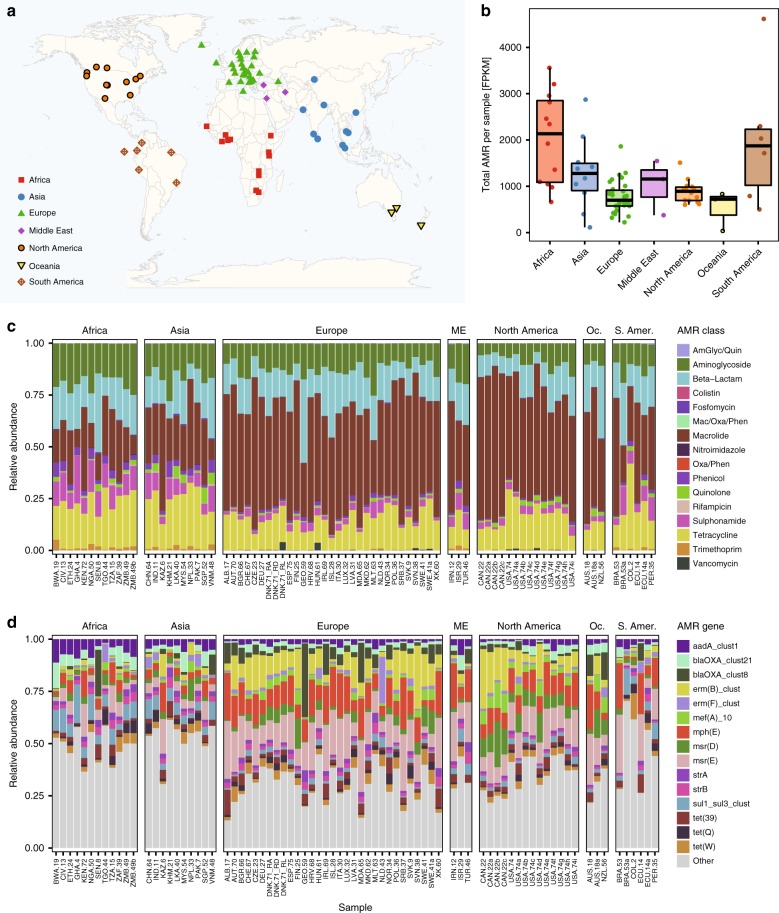
Global sewage sampling sites and overview of antimicrobial resistance (AMR) abundance and composition. **a** Map of the sampling sites. **b** Boxplots of the total AMR fragments per kilo base per million fragments per sample, stratified by region. Each sample is represented by a dot with horizontal jitter for visibility. The horizontal box lines represent the first quartile, the median, and the third quartile. Whiskers denote the range of points within the first quartile − 1.5× the interquartile range and the third quartile + 1.5× the interquartile range. **c** Relative AMR abundance per antimicrobial class (AmGlyc aminoglycoside, Mac macrolide, Oxa oxazolidinone, Phen phenicol, Quin quinolone). **d** Relative abundance of the 15 most common AMR genes (mef(A)_10: mef(A)_10_AF376746)

Domestic sewage was collected from 79 sample locations, covering 7 geographical regions from 74 cities in 60 countries (Fig. 1a, Supplementary Data 1). Each sample was sequenced using Illumina HiSeq and the resulting data (>1.4 Tb) processed using MGmapper^17^. The average number of reads per sample was 120 million reads (range: 8 million –398 million). An average of 0.03% of the reads were assigned to AMR genes, while on average 29%, 1%, 0.4%, and 0.2% were assigned to bacterial, protozoa, plants, and human genomic material, respectively (Supplementary Data 2). Sixty-eight percent of the reads could not be assigned to any reference sequence, and other metagenomic studies have also found larger number of un-mapped reads (42%–48%)^14,18^. Rarefaction of the reads mapping to bacterial genomes showed a tendency toward saturation in the sequence data (Supplementary Fig. 1).

Analyses of duplicate samples from separate days from eight sites showed a high degree of within-site reproducibility (Supplementary Fig. 2). Comparison of the data from the same countries showed much less (permutation test, *p* < 0.0001) variance across sites within countries than across sites between different countries (Supplementary Fig. 3), which suggests that a single sample taken from one large city is representative of the overall occurrence of AMR in a country.

A total of 1546 genera were detected across all samples (range: 942–1367 genera per sample), but a limited number of bacterial genera dominated (Supplementary Data 3, Supplementary Fig. 4). Several of the dominant bacterial genera were typical fecal, such as *Faecalibacterium*, *Bacteroides*, *Escherichia*, *Streptococcus*, and *Bifidobacterium*. However, other highly abundant bacterial genera, such as *Acidovorax* and *Acinetobacter*, are most likely environmental bacteria. Thus, the bacterial composition of sewage is complex and does not only reflect human feces but also the changes occurring in the sewer. A comparison with publicly available metagenomic data, although generated using different DNA-purification methods, suggested that our urban sewage samples resemble more the human fecal microbiome than the animal fecal microbiome from chickens, pigs, or mice (Supplementary Fig. 5).

The total AMR gene abundances varied across sites and continents (Fig. 1b, Supplementary Data 4). The highest AMR gene levels were observed in African countries (average: 2034.3 fragments per kilo base per million fragments (FPKM)), although Brazil had the highest abundance of all (4616.9 FPKM). At the lower end of the spectrum were Oceania (New Zealand and Australia) (average: 529.5 FPKM). To the best of our knowledge, comparable data on the global occurrence of AMR genes of predominantly healthy people do not exist. In agreement with our findings, a previous study on the resistome from toilet waste from long-distance flights^14^ suggested that the AMR levels in South Asia were higher than in Europe. Data on AMR in bacteria isolated from clinical infections in humans, collected by the World Health Organization (WHO)^1^, suggest a high prevalence of AMR in many developing countries, even though several of the national studies reported by the WHO give contradictory results^1^.

A total of 1625 different AMR genes belonging to 408 gene groups were identified, including several that have emerged recently, such as CTX-M, NDM, *mcr*, and *optrA* (Supplementary Data 4). Several different AMR genes might encode resistance to the same antimicrobial agent. Thus the relative abundance of AMR genes was aggregated to the corresponding antimicrobial class level for each sample to explore major trends across countries (Fig. 1c). AMR genes encoding resistance toward macrolides, tetracyclines, aminoglycosides, beta-lactams, and sulfonamides were the most abundant. Most samples from Europe and North America had a high relative proportion of macrolide resistance genes, while Asian and African samples had a large proportion of genes providing resistance to sulfonamides and phenicols. Fifteen AMR genes contributed >50% of the total AMR abundance (Fig. 1d). This proportion was especially prominent for Europe, North-America, and Oceania. None of the dominant AMR genes are known to be restricted to specific bacterial genera^19–21^.

### Global diversity and clustering of AMR genes

**Fig. 2:**
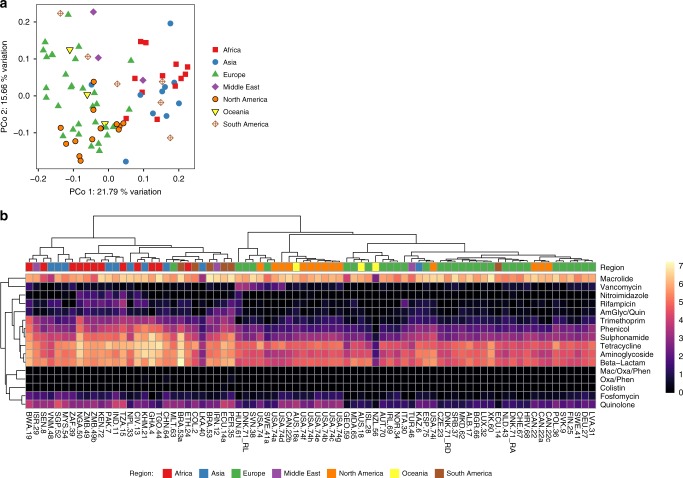
Resistome clustering in sewage samples across regions. **a** Principal coordinate analysis (PCoA) performed on the resistome Bray–Curtis dissimilarity matrix. The amount of variation explained by coordinates 1 and 2 is included in the axis labels. **b** Antimicrobial resistance class-level heat map. Relative abundances of genes (fragments per kilo base per million fragments (FPKM)) were summed to drug classes (AmGlyc aminoglycoside, Mac macrolide, Oxa oxazolidinone, Phen phenicol, Quin quinolone). Colors represent log (ln) transformed relative abundances (FPKM). Complete-linkage clustering of Pearson correlation coefficients was used to hierarchically cluster both samples and drug classes

We analyzed the AMR abundances on both the gene and antimicrobial class levels using both principal coordinate analyses (PCoAs) and heat maps (Fig. 2, Supplementary Fig. 6). With regard to the sample resistome dissimilarities, there was a clear geographical separation along the first principal coordinate of samples from Europe/North-America/Oceania and samples from Africa/Asia/South-America. Regional groupings explained 27% of the dissimilarity between sample resistomes (adonis2, *p* < 0.001; Fig. 2a). The separation among the groups was mainly driven by higher levels of resistance to tetracycline, aminoglycosides, beta-lactams, sulfonamides, and trimethoprim in the Africa/Asia/South America cluster, whereas macrolide resistance was more evenly distributed among all samples (Fig. 2b). A clearer clustering was observed based on regions, compared with clustering based on diet, income, or the Human Development Index (HDI) (Supplementary Figs. 6–7). A stronger regional separation was observed on the AMR class level, compared with the gene level (Fig. 2b, Supplementary Fig. 6). On the class level, a very clear separation of all samples in two groups was observed, where only a single European sample (Malta) did not cluster with all other samples from Europe/North America/Oceania (Fig. 2b). Furthermore, only one sample from Asia (Kazakhstan), one from the Middle East (Turkey), and one from South America (Galapagos Islands) clustered with the Europe/North-America/Oceania group.

Several alpha diversity indices for each sample resistome were calculated (Supplementary Fig. 8). The resistome median evenness was higher in African and Asian countries compared with resistomes from other geographical locations. Thus not only do these regions have a higher prevalence of AMR genes, but also a more equal distribution of the different AMR genes.

Analyses of the alpha diversity of the bacterial taxonomic composition showed less separation according to geographical regions compared with the AMR genes (Supplementary Figs. 9–11), and regions explained less of the variation in the resistome (adonis2, 27%) compared with the bacterial composition (adonis2, 17%). To test the degree to which bacterial genus-level composition of the microbiota is associated with the resistomes, Procrustes analyses were performed (Supplementary Fig. 12). We compared ordinations of the bacterial taxonomic composition with the resistome and found that they correlated significantly (protest, *p* < 0.001).

### AMR genes and drug use association

Several studies have shown that antimicrobial use (AMU) selects for AMR^22,23^ and that reducing AMU often results in reduced AMR^24,25^, except when there is interference between genetically linked AMR genes conferring resistance to different antimicrobial classes^26^. It has also been suggested that AMU explains only some of the variation^22,23^ and that other factors such as diet, cultural traditions and occupation also have an influence^22,27^.

In this study, the association between AMU and the occurrence of AMR in the sewage samples was estimated using a generalized linear mixed-effects model, with the counts of genes in the different antimicrobial classes as an outcome (Poisson) adjusting for sequencing depth and gene length. As AMU data, we included 2015 data from Europe [www.ecdc.dk] and IQVIA, formerly Quintiles IMS Holdings, Inc. (see Methods). In the regression model, we accounted for the potential effects of cross-resistance by fitting fixed effects of both usage of the antimicrobial class that a resistance gene primarily confers resistance to (direct selection for resistance) and the total AMU (indirect selection via cross-resistance). While our model showed a significant increase in the abundance of AMR genes belonging to a specific antimicrobial class with increasing usage of that antimicrobial class, we found no significant effect of total usage of all antimicrobials on abundance of the different classes (Supplementary Fig. 13A, Supplementary Table 1). This suggests that, while AMU of a specific class is an important driver of AMR genes encoding resistance to that class, the effects of cross- and/or co-resistance appear to have a relatively minor contribution to AMR abundances. Furthermore, our model showed that the countries with a lower HDI (i.e., higher in rank) have lower abundances of AMR genes (glmm, *p* = 0.01) and that the number of passengers arriving in a country via flights has no effect on the abundance of AMR genes (glmm, *p* = 0.62).

A second (similar) model was developed to test the association between the abundance of AMR genes on the antimicrobial class level and the antimicrobial residue levels. Again we accounted for the potential effects of cross-resistance by fitting fixed effects of both residues of the antimicrobial class that a resistance gene primarily confers resistance to (direct selection for resistance) and the total antimicrobial residue levels (indirect selection via cross-resistance). As before, this model showed a significant increase in the abundance of AMR genes belonging to a specific antimicrobial class with increasing levels of drug residues of that antimicrobial class; however, we found no significant effect of total residue levels (Supplementary Fig. 13B). As with the previous model, this model also showed that the countries with a lower HDI (i.e., higher in rank) were less abundant in terms of AMR genes (glmm, *p* < 0.001), and that the number of passengers arriving in a country via flights had no effect on the abundance of AMR genes (glmm, *p* = 0.746), indicating that the results are robust.

Of interest is that there was no correlation between AMU and antimicrobial residue in our data (lm, R2 =  0.00098, *p* = 0.55). One possible explanation for this could be that AMR abundances accumulates on a long timescale because of the average usage in a country (i.e., (long term) AMU impacts the level of resistance); however, on a much shorter timescale fluctuations in AMU will change the AMR composition (diversity) somewhat, which is captured by the residue levels, which can be interpreted as a (short) temporal snapshot of the actual usage.

### Prediction of AMR based on population-level health data

We observed that AMU only explained a minor part of the occurrence of AMR across the world. In addition, AMU data are difficult to obtain and likely subject to limitations due to the lack of an effective prescription system in many countries. Measuring antimicrobial residue levels in sewage as a proxy for AMU is also associated with uncertainties and the rapid degradation in the environment of the heavily used beta-lactamase-sensitive antimicrobials makes it difficult to reliably measure them.

**Fig. 3:**
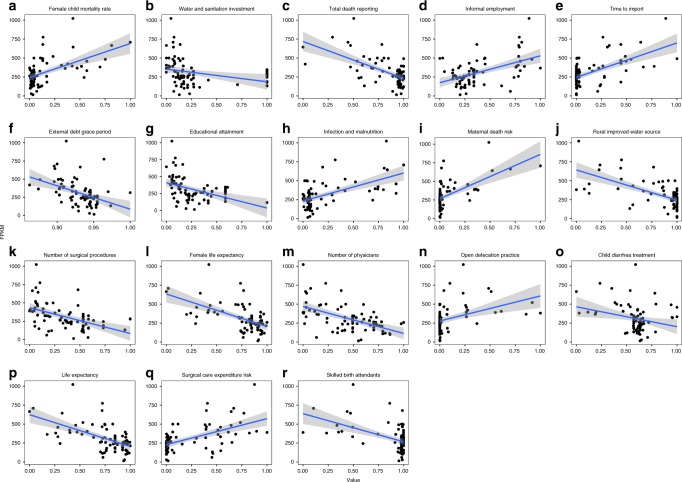
World Bank variables significantly associated with the observed antimicrobial resistance abundances. Detailed information concerning the variables in **a**–**r** are presented in the same order in Supplementary Table 3

**Fig. 4:**
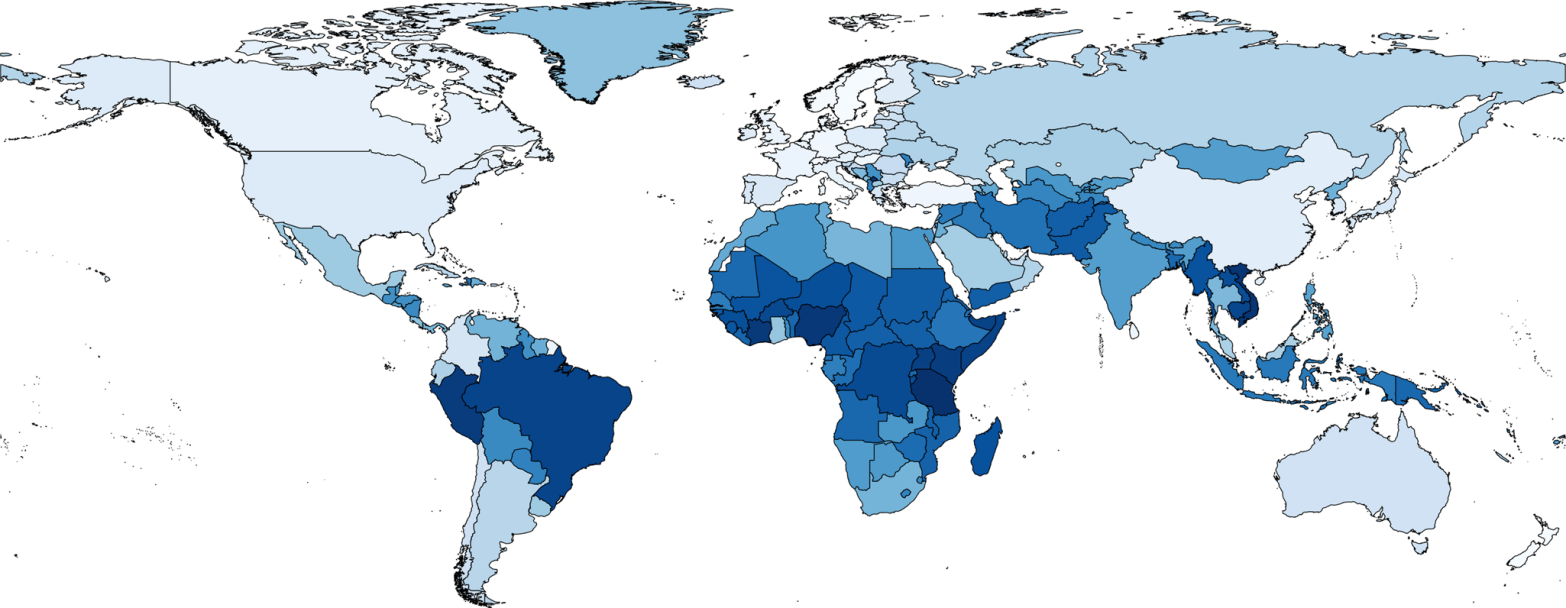
Global predictions of antimicrobial resistance (AMR) abundance in all countries and territories in the world. Map colored according to predicted abundance of AMR from light blue (low AMR abundance) to dark blue (high AMR abundance). Global resistance predictions for the 259 countries and territories are shown in Supplementary Data 5

Because the HDI was strongly associated with AMR, in the results of our model, we hypothesized that a number of other factors could be either drivers or indicators for AMR. To investigate this further, we used 1503 variables from the World Bank’s Health, Nutrition and Population as well as Development indicator data sets collected between the years 2000 and 2016 for 259 countries and territories to study the potential association with the observed AMR gene abundances. Using country-specific variables, we were able to explain up to 89% of the observed variation across the samples (Supplementary Fig. 14, Supplementary Table 2). Most of the variables associated with AMR levels were related to sanitation and general health (Fig. 3, Supplementary Fig. 15, Supplementary Table 3). Subsequently, the identified variables were used to predict the occurrence of AMR in 259 countries and territories. The three countries predicted to have the lowest level of AMR were The Netherlands, New Zealand, and Sweden, whereas the highest predicted AMR levels were for Tanzania, Vietnam, and Nigeria (Fig. 4, Supplementary Data 5). The predicted global country-level resistance levels were multiplied with the latest national population estimate and used to create global maps of healthy human-associated AMR (Supplementary Fig. 16).

## Discussion

A reliable base of evidence that accurately describes and characterizes the global burden and transmission of AMR is essential to support national and global priority setting, public health actions, and treatment decisions. Compared to samples directly obtained from humans, a major advantage of sewage is that such samples can be easily obtained and analyzed without ethical concerns. Thus, while current efforts to improve surveillance of AMR in human clinical pathogens should be continued^1,3–5^, we do suggest that our study provides the foundation for a flexible, simple, affordable, and ethically acceptable global real-time surveillance of AMR that could be immediately implemented globally also in low- and middle-income countries. The study design can furthermore be used for other infectious disease agents.

Our study represents, to the best of our knowledge, the first attempt to monitor and predict the occurrence of AMR in the global predominantly healthy human population. Even though the study suffers from the limitation that only a single sample was analyzed from each site, our study suggests a strong systematic separation of regions of the world according to AMR gene abundance with high-income countries in Europe/North-America/Oceania constituting one cluster and low-income countries in Africa/Asia/South-America constituting another cluster. This separation was mainly driven by a relatively high abundance of a limited number of AMR genes encoding macrolide resistance genes in Europe/North-America/Oceania, compared with a general high abundance of several different AMR genes from different classes in the rest of the world. The countries standing out as having the most divergent distribution of AMR genes were Vietnam, India, and Brazil, suggesting that these countries could be hot spots for emergence of novel AMR mechanisms.

In this study, we used metagenomics that benefit from the ability to quantify thousands of genes simultaneously and that the data can be reanalyzed if novel genes of interest are identified. However, other methodologies might also be useful such as culture and PCR-based methods that might have better sensitivity. Comparative studies evaluating the usefulness of various technologies, including evaluation of sensitivity, specificity, and number of targets detected, are warranted.

In our study, we have focused on total AMR abundance. However, the resistance to the different antimicrobial classes are not equally important^28^ neither are all AMR genes^29^. Thus further studies could also benefit from addressing specific resistance genes.

An analysis of the same IQVIA AMU data from 2000 to 2015, as we have used here, was recently published^30^, and even though a significant increase in AMU was observed, especially for countries in Africa, Asia, and South-America, the quantities are still below most countries in Europe and North-America. We could not in our study find significant associations between obtainable AMU data and the concentrations of residues measured, but we did observe the highest concentrations of antimicrobial agents in the African samples, which could suggest that measuring residues in sewage could provide alternative data for monitoring AMU compared to obtainable sales data.

Historically, most strategies to reduce AMR have focused on reducing AMU, which relies upon AMR imparting a fitness cost on the bacterial host, an effect that has been relatively weak in horizontally transferred AMR genes compared with chromosomal mutations^31^. Other factors, such as those related to transmission, including infection control, sanitation, access to clean water, access to assured quality antimicrobials and diagnostics, travel, and migration, have also been suggested to contribute significantly to AMR^27^. In this study, we found that, irrespective of the diversity of AMR genes, the total AMR abundance was highly correlated with a limited number of World Bank variables, mainly concerning sanitation and health. In contrast, human air travel had no significant influence on AMR abundance. Importantly, this suggests that the total AMR abundance is mainly influenced by local/national parameters, and even though all AMR genes might rapidly disseminate and be found in all corners of the world, local selection is required for them to reach appreciable frequencies. These findings suggest that improving sanitation, health, and perhaps education as part of the Sustainable Development Goals [www.un.org/sustainabledevelopment/sustainable-development-goals/] would be effective strategies for limiting the global burden of AMR.

## Methods

### Ethics

It was confirmed that this and similar studies using human sewage in accordance with the Danish Act on scientific ethical treatment of health research (Journal no.: H-14013582) do not require preapproval from ethical committees.

### Collection of urban sewage samples

The National Food Institute, Technical University of Denmark (DTU Food) launched an open invitation 25 September 2015 seeking potential collaborators to participate in the pilot study of the Global Sewage Surveillance Project (GSSP). The invitation was sent by electronic mail to the following networks and individual research collaborators for further dissemination: WHO Global Foodborne Infections Network (GFN) [http://www.who.int/gfn/en/], WHO Advisory Group on Integrated Surveillance of Antimicrobial Resistance (AGISAR) [http://www.who.int/foodsafety/areas_work/antimicrobial-resistance/agisar/en/], European Union Reference Laboratory for AMR network [www.eurl-ar.eu/], and to the European Food- and Waterborne Diseases and Zoonoses Network (FWD-Net) [https://ecdc.europa.eu/en/about-us/partnerships-and-networks/disease-and-laboratory-networks/fwd-net].

Participation was cost neutral and managed by online registration that also included requests for proper packaging and sample containers and the need to sign a material transfer agreement or similar approval/agreement to protect any intellectual property rights. All expenses related to the shipments were paid by DTU Food, and the shipments complied with the IATA regulation as per SP A197 because the content of the parcels was identified as UN3082 “Environmentally hazardous substance, liquid, not otherwise specified” but did not exceed 5 L of sewage.

A protocol that instructed how to collect the urban sewage samples as well as epidemiological, demographic, and geographical information was provided to each participant in the study (Supplementary Data 8). Participants were requested in conjunction with collecting the samples to submit via an online survey the captured information related to epidemiological, demographic, and geographical information as well as a digital image of the sampling site, if possible the GPS coordinates of the sampling location, the temperature of the sample at the time of sampling, pH of the sewage, and storage temperature of sample. From each location, on 2 consecutive days between 25 January and 5 February 2016, one representative, non-processed, unfiltered urban sewage sample of 2 L was collected from the respective main sewage pipeline(s) prior to the inlet of the wastewater treatment plant or from the main outlet to rivers or similar according to the protocol provided. Flow proportion sampling over 24 h was preferred. Alternatively, three crude point samples were collected in a short time interval, i.e., at least 5 min between each individual sample, to ensure as much randomness as possible. The collected urban sewage was not treated, neither with additives nor DNA stabilizers and was recommended to be stored at −80 °C for at least 48 h prior to shipment to avoid the use of dry ice. The sample-specific data are provided in Supplementary Data 1 and an explanation of the content of this file provided as Supplementary Note 1.

### Sample handling and DNA extraction

At DTU Food, a photograph of each sample container was taken upon arrival combined with a short remark describing the condition of the sample, e.g., solid frozen, thawed, coloration, etc. The samples of the first of the consecutive days were thawed for 12 h at approximately 20 °C before processing. After thawing, 250 mL of each sample were pelleted in a centrifuge at 10,000 × *g* for 10 min. The pellet was stored at −20 °C or −80 °C before DNA extraction and metagenomics analysis, and the supernatant was stored at −80 °C for subsequent antimicrobial residue and virus analysis. DNA was extracted from the sewage pellets according to an optimized protocol using the QIAamp Fast DNA Stool Mini Kit including twice the input material and initial bead beating^32^. For each batch of DNA extractions, a DNA extraction blank control was processed in parallel with the sewage samples to monitor background DNA.

### Whole-community sequencing

DNA was shipped on dry ice for library preparation and sequencing to the Oklahoma Medical Research Foundation (OMRF). DNA from all samples was mechanically sheared to a targeted fragment size of 300 bp using ultrasonication (Covaris E220evolution). Library preparation was performed with the NEXTflex PCR-free Library Preparation Kit (Bioo Scientific). The Bioo NEXTflex-96 adapter set (Bioo Scientific) was used, and in batches of roughly 60 samples, the libraries were multiplexed and sequenced on the HiSeq3000 platform (Illumina), using 2 × 150-bp paired-end sequencing per flow cell. The raw sequencing data have been deposited at the European Nucleotide Archive under accession number ERP015409.

### Trimming and mapping of sequencing reads

The reads were trimmed, including adaptor removal, using BBduk [BBMap—Bushnell B.—https://sourceforge.net/projects/bbmap/] with a quality threshold at 20 and minimum length of 50 bp. Trimmed reads were used as input to the reference-based mapping and taxonomy-assignment tool MGmapper^17^, which is based on BWA-MEM^33^ version 0.7.12 and SAMtools^34^ version 1.6. Reads were aligned against reference sequence databases for the best hit (Bestmode, i.e., a read can only map to 1 reference sequence). An acquired AMR gene database (ResFinder)^35^ was used to annotate properly paired reads (MGmapper option fullmode) where each read pair had sequence coverage of at least 80% compared with the length of the trimmed reads. This was the only filter that was applied to discard a read pair. The AMR genes were of bacterial origin and could therefore align to both bacteria databases and the Resfinder database. To enable multiple database hits, AMR genes were mapped using the Fullmode option in MGmapper for the most optimal abundance calculation. Genomic annotation was performed by identifying the best hit (MGmapper option bestmode) for a pair of reads when aligned against a range of reference sequence databases. Databases were primarily downloaded via NCBI genbank clade specific assembly_summary.txt files unless another ftp site is provided below. The list of databases used by MGmapper includes: Human (GRCh38.p3), bacteria (closed genomes), MetaHitAssembly (PRJEB674—PRJEB1046), HumanMicrobiome (genomes assemblies), bacteria_draft, plasmid, archaea, virus, fungi, protozoa, vertebrates_mammals, vertebrates_other, invertebrates, plant, and nt. For the bacteria and bacteria_draft databases, sequences were selected from the assembly_summary.txt file, when annotated with the tags version_status=‘latest’ and genome_rep=‘Full’. Furthermore, assembly_level= ‘Complete genome’ or ‘Chromosome’ were required for entries in the bacteria database and refseq_category=‘representative genome’ for entries in the bacteria_draft database. The plasmid database was constructed as the subset of bacteria and bacteria_draft sequences having the word ‘plasmid’ in the fasta entry header line.

The total bacteria read count for a sample was calculated as the sum of read counts from each of the bacteria-related databases (bacteria, bacteria_draft, MetaHitAssembly, and HumanMicrobiome). The total fraction of unmapped reads for all sample sites were used to translate the percentage of unmapped reads into *Z*-scores, i.e., the number of standard deviations from the mean. Setting an absolute *Z*-score threshold at 3 retained data from 79 sample sites. Data from Chad were excluded based on the *Z*-score threshold together with data from Gambia with suspiciously low resistance read counts; i.e., lower than those observed from DNA extraction control samples.

### Bacterial and AMR gene distribution

Inspection of the count tables and the mapped reads distribution on the genomes revealed an overestimation of some genomes due to plasmids with high copy numbers. The issue only occurred in the included draft genomes, as the plasmid DNA were left out of the database with complete bacterial genomes. The distribution of mapped reads across contigs was expected to be evenly distributed, with some variation. The plasmids were revealed by large hit counts to specific contigs compared with the associated contigs in a draft genome. For each draft genome, the hits to each contig was normalized with respect to contig size. The median of the contig hits was found and the third quartile and the interquartile range was calculated. If a hit count was above the third quartile plus 1.5 times the interquartile range, then the hit count was interpreted as an overestimation and adjusted by replacing the hit count with the median.

Relative abundance of AMR genes and bacterial genera were calculated as FPKM. This was done to account for both sample-wise sequencing depth differences and size-dependent probability of observing a reference. For bacterial genome assemblies, FPKM was calculated based on the adjusted sum of fragments assigned to a genome assembly, whether or not the genome was closed. For AMR genes, FPKM was calculated on each individual ResFinder reference sequence. FPKMs were subsequently summed up across categories to bacterial genera (NCBI taxid), drug class level (NCBI tax ID), and highly homologous AMR gene groups (CD-HIT-EST, 90% similarity)^36^.

### Within-site reproducibility

In order to test reproducibility of sewage samples, a dendrogram of resistome composition from all sewage samples including samples from eight sites that were double-sampled sewage was generated using Bray–Curtis (BC) dissimilarities and hierarchical clustering (Supplementary Fig. 2). The replicated samples were taken by 2 days apart. The day 2 samples were taken twice. The day 2 samples were kept in freezer for 2 years prior sequencing. Samples from day 1 and day 2 were sequenced using the same DNA extraction and sequencing protocols. The replicated samples had higher resistome similarity to its own replicates and all eight sets of replicate samples were clustered with their own replicate. This result shows a strong reproducibility of sewage samples despite having different day of sampling and 2 years of storage. The repeats were not included in subsequent analyses.

### Within-country representativeness

To assess whether samples from individual sites are representative of other sites in that country, we compared the BC dissimilarities for pairs of sites within the same country and in different countries for both resistome and bacteriome compositions. We assessed the significance of these differences using permutation tests. We permuted the country labels for each sample and reassessed the dissimilarities for pairs of sites within the same country and in different countries with permuted labels. We repeated this procedure 10^6^ times to build up a null distribution of the differences in dissimilarity within and among countries. We found that resistome dissimilarities were on average 34% higher for pairs of sites in different countries than for pairs of sites within the same country (permutation, *p* < 0.0001, Supplementary Fig. 3A and B), while bacteriome dissimilarities were 46% higher for pairs of sites in different countries than for pairs of sites within the same country (*p* < 0.0001, Supplementary Fig. 3C and D). These results show that there is less variance across sites within countries than across sites within different countries. Thus individual sites in this study are representative of other sites in that country.

### Sample composition comparison

Metagenomes with the following accession numbers were downloaded from SRA (December 2017): ERR011089, ERR011114, ERR011344, ERR1104480, ERR1104481, ERR1121453, ERR1121455, ERR1121556, ERR1135427, ERR1135431, ERR1135437, ERR1135693, ERR1278103, ERR1278104, ERR1278105, ERR1414230, ERR1414260, ERR1527239, ERR1527247, ERR1558700, ERR1559789, ERR1560016, ERR1560024, ERR1560100, ERR1655116, ERR1682090, ERR1682101, ERR1698980, ERR186217, ERR1950597, ERR1950599, ERR1950601, ERR469632, ERR469644, ERR469650, ERR675524, ERR675555, ERR675557, ERR675560, SRR1182511, SRR1202091, SRR1267595, SRR2175658, SRR2175725, SRR2175750, SRR2751194, SRR2891615, SRR2891618, SRR605600, SRR605634, SRR873603, SRR873608, and SRR924749. The quality of the data was assessed and if necessary adapters were removed and trimmed with a quality threshold at 20 and minimum length of 50 bp. All pairwise Jaccard distances of the aforementioned metagenomes, all global sewage samples, and the control samples were calculated with Mash^37^. The heat map was drawn in R 3.4.4 (*pheatmap)*.

### Sample dissimilarities

Matrices with relative abundances (FPKM) of AMR genes and bacterial genera were Hellinger-transformed using the *decostand* function in vegan. The BC dissimilarity matrices were then calculated using the *vegdist* function, also in vegan (Supplementary Data 6).

### Heat maps

The relative abundances (FPKM) of AMR genes were log-transformed and visualized in a heat map (*pheatmap*), showing the 50 most abundant genes. The gene-wise dendrogram is based on Pearson product moment correlation coefficients (PPMC), while the sample dendrogram is based on the aforementioned BC dissimilarity matrices, not just for the visualized genes, but all genes. Both dendrograms are the result of complete linkage clustering. The bacterial genera heat map was produced in the same way, while the AMR class-level heat map differs by using PPMC for hierarchical clustering of both AMR classes and samples. Continent association was included as metadata for all heat maps. For the AMR genes, we included World Bank income levels [http://apps.who.int/gho/data/node.metadata.COUNTRY?lang=en], GEMS cluster (identifies countries with similar dietary intake)^38^, the HDI^39^, and GBD 2015 super-regions^40^.

### Sample ordination

Dissimilarity matrices were subject to classical multidimensional scaling (PCoA) to obtain the first two principal coordinates as well as the variance explained by each, ignoring negative eigenvectors. This was done using the cmdscale command in R.

### Testing of sample dissimilarities

The BC dissimilarity matrices used for PCoA were also used for permutational multivariate analysis of variance (adonis2 function in vegan). The geographical group assigned to each sample was used as a predictor for dissimilarity.

### Alpha diversity

Diversity and richness were calculated on rarified versions of the resistance and bacterial count matrices. For resistance genes, the two samples with <1000 read pairs were excluded. Subsequently, count matrices were subsampled to the lowest samples’ depth, using the Vegan rarefy function. The Simpson diversity index (1-D), Pielou’s evenness, and the Chao1 richness estimates were calculated using the diversity function in the vegan package.

### Procrustes analysis

The vegan package was used for comparing the resistome dissimilarities with the bacteriome dissimilarities. The protest function was used to scale and rotate the principal coordinates of the bacterial PCoA onto the principal coordinates of the resistome PCoA and testing the strength of the association.

### Graphics and statistics

All plots and statistical analyses were carried out in Microsoft R Open 3.3.2.

### Correlation between AMU and external explanatory variables

A multilevel Poisson model was developed to investigate the sources of variance for the abundance of AMR genes and the relationship between AMU and abundance of AMR genes found in the samples. The counts of the individual AMR genes in each of the samples (see “Collection of urban sewage samples” and “Whole-community sequencing“ sections) aggregated at the antimicrobial class level was used as the dependent variable.

An observation-level random effect was used to model the over dispersion inherent to count data^41^. Because several samples were sequenced more than once on separate sequencing runs, we were able to estimate and correct for the noise in mean AMR gene abundance owing to the sampling process. Therefore, a categorical variable indicating which sample was used was included as a random effect (sample). A categorical variable identifying each location to allow for the estimation of variation in abundance of resistance genes between the different sampling locations (location) was included. Furthermore, a variable identifying the resistance class a resistance gene is a member of was included to estimate the variance due to differences in abundance between antimicrobial classes (class).

For the fixed effects, we included a measure of AMU at the country level. Data from the ECDC database (Supplementary Data 1) and data from the IMS database (Supplementary Data 1) were used to calculate a new variable from the two data sets. To this end, we used the data from the ECDC data set where available to predict missing values from the IMS data set by using a linear regression model. Because the data from the two data sets are highly correlated (*r* = 0.97, *p* < 0.01, Pearson’s product moment correlation), we could infer the (approximate) corresponding ECDC value from the IMS value for that particular country. The antimicrobial usage data were then log-transformed. Because it has been argued that many antimicrobial classes show cross-resistance, where resistance to one antimicrobial class also confers resistance to another class, we accounted for the potential effects of cross-resistance by fitting effects of both usage of the antimicrobial class that a resistance gene primarily confers resistance to (direct selection for resistance) and total AMU (indirect selection via cross-resistance). We also included the total number of passengers arriving in a country in 2015 as a fixed effect in the model. Data on the number of passengers were extracted from the ICAO international flight database [https://portal.icao.int, downloaded April 2016] and log-transformed. Lastly, we included the United Nations HDI as a fixed effect. HDI data from 2015 were extracted for the United Nations Development Programme website^39^ and log-transformed and scaled before including them in the final model. The same model set-up was used to investigate the association between drug residue levels and AMR gene abundance. In this model, data on the drug residues in the samples was included as a fixed effect instead of the AMU data. All other effects (random and fixed) were kept the same. Others models including temperature at the collection site at the day of sampling and Gross Domestic Product showed no significant association with AMR genes abundance (glmm’s, all *p* > 0.05).

### Resistance prediction

World Bank Health, Nutrition and Population as well as Development indicator data sets collected between the years 2000 and 2016 for 259 countries and territories were downloaded from http://databank.worldbank.org/data/home.aspx in October 2017 and used to formulate AMR predictive models. Imputations of missing data were conducted using the *missForest* R package, which is a random forest-based technique that is highly computationally efficient for high-dimensional data consisting of both categorical and continuous predictors^42^. The final data set consisted of 1503 economic and health indicator variables (Supplementary Data 7). The most important variables predicting total resistance (FPKM) were selected from the World Bank data set and a recursive feature elimination method from the R library caret (Supplementary Table 3). The model was also run on all 1503 variables for comparison (Supplementary Table 4) and a good correlation was observed. The machine learning algorithms Support Vector Machine and random forest were compared for accuracy in predictions based on their R^2^ and Root Mean Square Error^43,44^. Random forest was the best choice of model (Supplementary Table 2). Random forest is suitable for data sets with many features, especially where each of the features contributes little information^45^. The prediction model was trained for the 60 countries where resistance data were available from the current project followed by global predictions of resistance for all 259 countries and territories (Supplementary Data 5).

Overfitting remains a major hurdle when applying predictive models especially involving many predictors. Breiman^45^ proved that random forest is protected from overfitting by the incorporation of out-of-bag (OOB) estimates and from the law of large numbers. This followed from earlier proposals on the use of OOB estimates as a key part of estimation of generalization error^46,47^. In the study of error estimates for bagged classifiers, Breiman^48,49^ provided empirical evidence demonstrating same accuracy from using the OOB estimate as using a test set of the same size as the training set and further indicated that the use of the OOB error estimate removes the need for a set aside test set. OOB is the mean prediction error on each training sample *x*_i_, using only the trees that did not have *x*_i_ in their bootstrap sample^50^. During random forest training, approximately one third of the instances are left out from each bootstrap training set. This means that the OOB estimates are computed from only about one third as many classifiers as in the ongoing main combination. However, the error rate decreases as the number of combinations increases, which means the OOB estimates will tend to overestimate the current error rate. To get unbiased OOB estimates, random forests are run past the point where the test set error converges. This has an added advantage that, unlike cross-validation, where bias is present but its extent unknown, the OOB estimates are unbiased^51^.

Random forests also aggregate many decision trees to limit overfitting as well as error due to bias because of the law of large numbers. Random forests limit overfitting without substantially increasing error due to bias due to their ability to mitigate the problems of high variance and high bias^45^.

However, a ten-fold cross-validation was included during our model building by randomly partitioning model input samples into ten sets of roughly equal size followed by estimation of accuracy based on held-out samples. This held-out sample was each time returned to the training set and the procedure was repeated with the second subset held out and so forth. Cross-validation and checking for valid accuracy were also performed with an implication that accuracy scores reduce if there is overfitting and only “valid accuracy” is finally used.

### Creation of global figures and maps

QGIS 2.18.11 using the cartogram package was used to create colored and distorted maps.

### Analysis of tetracyclines, sulfonamides, macrolides, and quinolone

The analysis of tetracyclines, sulfonamides, macrolides, and quinolones in the sewage samples were performed as in Beredsen et al.^52^, with adaptations as summarized in the following text. For pretreatment, two 10-mL portions of each sample of sewage supernatant were weighed into separate 50-mL tubes, after which internal standards were added. To one of these portions, antimicrobials were added at a level of 25 ng/L for the sulfonamides and 100 ng/L for the tetracyclines, quinolones, and macrolides. Four mL of EDTA-McIlvain buffer (0.1 M, pH 4.0) were added, after which the samples were shaken for 5 min head-over-head. The residue was taken up in 100 μL MeOH, after which 400 μL of water was added. For liquid chromatography tandem mass spectrometry (LC-MS/MS), the following gradient was applied: 0–0.5 min 1% B; 0.5–2.5 min linear increase to 25% B; 2.5–5.4 min linear increase to 70% B; 5.4–5.5 min linear increase to 100% B, with a final hold of 1.0 min. The injection volume was 5 μL. Detection was carried out by an AB Sciex (Ramingham, MA, USA) Q-Trap 5500 or a Q-Trap 6500 mass spectrometer in positive electrospray ionization (ESI). The parameters used for the Q-Trap 5500 and the Q-Trap 6500 were: capillary voltage, −4.0 kV; declustering potential, 10 V; source temperature, 450 °C; GAS 1 and 2, 50 (arbitrary units).

### Analysis of aminoglycosides

The analysis of aminoglycosides in the sewage samples was performed as in Bello^53^, with adaptations as summarized in the following. For sample pretreatment, two 10-mL portions of each sample of sewage were weighed into separate 50-mL tubes, after which internal standards were added. To one of these portions, aminoglycosides were added at a level of 50 μg/L. Twenty mL of extraction liquid (10 mM KH_2_PO_4_ with 0.4 mM EDTA and 2% TCA) were added, and samples were mixed by means of a vortex and shaken head-over-head for 10 min. The extract was then brought to pH 7.6–7.9 and centrifuged (15 min, 3600 × *g*). The complete extract was transferred to a conditioned CBX cartridge, followed by washing with 4 mL of water and drying. The aminoglycosides were eluted with 3 mL of acetic acid (10% in MeOH). The eluate was dried at 60 °C, evaporated under N_2_ and taken up in 400 μL of HFBA (0.065%). For LC-MS/MS, the following gradient was applied: 0–0.5 min, 0% B; 0.5–5 min, linear increase to 45% B; 5–8 min, linear increase to 60% B; 8–10 min, linear increase to 100% B. The injection volume was 40 μL. Detection was carried out by a Waters (Milford, MA, USA) Quattro Ultima mass spectrometer in positive ESI mode. The parameters used were: capillary voltage, 2.7 kV; desolvation temperature, 500 °C; source temperature, 120 °C; cone gas, 150 L/h; and desolvation gas 550 L/h.

### Analysis of β-lactams

The analysis of β-lactams in the sewage samples was performed as in Beredsen et al.^54^, with adaptations as summarized in the following. For sample pretreatment, two 10-mL portions of each sample of sewage were weighed into separate 50-mL tubes, after which internal standards were added. To one of these portions, β-lactams were added at a level of 50 μg/L for the penicillins and 500 μg/L for the cephalosporins and carbapenems. Detection was carried out by a Waters model Xevo TQS or an AB Sciex (Ramingham, MA, USA) Q-Trap 6500 mass spectrometer in positive ESI mode. The parameters used for the QTrap 6500 were: capillary voltage, 2.0 kV; cone voltage, 25 V; source offset, 20 V; source temperature, 150 °C; desolvation temperature, 550 °C; cone gas flow, 150 L/h; and desolvation gas, 600 L/h.

### Calculation of defined daily dosages (DDDs) based on residues

After consumption, antimicrobials undergo (1) metabolization in the body, (2) are eliminated from the body with urine and/or feces, and might (3) further degrade in the sewer. A proper calculation of DDD/person/day from concentrations in sewage therefore requires information on elimination rates and a precise estimate of the number of persons connected to a sewer and the total water flow at this specific location (or the average water consumption per person as surrogate). Here information on elimination rates and water consumption were not included. In addition, we can only calculate DDD for human drugs used for systematic infections. As seen from Supplementary Data 1, the amount of dihysdrostreptomycin is almost absent, the use of Dapson very low, and the contribution of enrofloxacin and tylosin 0.1% and 0.3% of the residues of quinolones and macrolides, respectively. For the sulfonamides, the excluded drugs constitute 11%.

The concentrations of antimicrobial residues were transformed into DDDs of antimicrobials for systemic use using the WHO DDD database [www.whocc.no/atc_ddd_index]. Some residues were excluded as their predominant usage was not systemic. This was the case for Dapson (main treatment indication: Lepra treatment), sulfacetamide (main indication: acne), sulfadoxine (main indication: malaria), sulfathiazole (main indication: topical application, with varying dosages), enrofloxacin (animal use—no WHO/ATCC DDD available), tylosin (animal use—no WHO/ATCC DDD available), and dihydrostreptomycin (animal use—no WHO/ATCC DDD available). The DDD were summed over the respective antimicrobial classes. Calculation of the sum of DDD across classes was not deemed meaningful, because excretion rates differ largely between antimicrobial classes and also within antimicrobial classes.

## Supplementary information

Supplementary Information

Peer Review File

Description of Additional Supplementary Files

Supplementary Data 1

Supplementary Data 2

Supplementary Data 3

Supplementary Data 4

Supplementary Data 5

Supplementary Data 6

Supplementary Data 7

Supplementary Data 8

## Data Availability

The raw sequencing data have been deposited at the European Nucleotide Archive under accession number ERP015409. Data on the number of passengers were obtained under license from the ICAO international flight database and is available from the authors with restrictions. Data on antimicrobial use were obtained from ECDC and IMShealth and the data used in our analyses are provided in supplementary Table 1. IMShealth data were obtained under license and has restricted use. All source data underlying the multivariate analyses, the machine learning and Figs. 1–4 and supplementary Figs. 1–16 are included in supplementary tables or supplementary data.

## References

[1] World Health Organization. *Antimicrobial Resistance: Global Report on Surveillanc*e. [https://apps.who.int/iris/bitstream/10665/112642/1/9789241564748_eng.pdf] (WHO Press, World Health Organization, Geneva, 2014).

[2] Aarestrup FM (2015). The livestock reservoir for antimicrobial resistance: a personal view on changing patterns of risks, effects of interventions and the way forward. Philos. Trans. R. Soc. Lond. B Biol. Sci..

[3] Zignol M (2016). Twenty years of global surveillance of antituberculosis-drug resistance. N. Engl. J. Med..

[4] Weston, E. J., Wi, T. & Papp, J. Strengthening global surveillance for antimicrobial drug–resistant *Neisseria gonorrhoeae* through the Enhanced Gonococcal Antimicrobial Surveillance Program. *Emerg. Infect. Dis*. **23**, 10.3201/eid2313.170443 (2017). 10.3201/eid2313.170443 PMC5711314 29155673

[5] Hay SI (2018). Measuring and mapping the global burden of antimicrobial resistance. BMC Med..

[6] United Nations, Department of Economic and Social Affairs, Population Division (2014). *World Urbanization Prospects: The 2014 Revision, Highlights (ST/ESA/SER.A/352)* (WHO Press, World Health Organization, Geneva).

[7] UNICEF/WHO. Progress on Sanitation and Drinking Water – 2015 Update and MDG Assessment. United Nations. [https://esa.un.org/unpd/wup/publications/files/wup2014-highlights.pdf] (accessed April 2018).

[8] Baum R, Luh J, Bartram J (2013). Sanitation: a global estimate of sewerage connections without treatment and the resulting impact on MDG progress. Environ. Sci. Technol..

[9] Fernández MD (2012). Environmental surveillance of norovirus in Argentina revealed distinct viral diversity patterns, seasonality and spatio-temporal diffusion processes. Sci. Total Environ..

[10] Madico G (1996). Active surveillance for *Vibrio cholerae* O1 and vibriophages in sewage water as a potential tool to predict cholera outbreaks. J. Clin. Microbiol..

[11] Asghar H (2014). Environmental surveillance for polioviruses in the Global Polio Eradication Initiative. J. Infect. Dis..

[12] Hovi T (2012). Role of environmental poliovirus surveillance in global polio eradication and beyond. Epidemiol. Infect..

[13] Munk P (2017). A sampling and metagenomic sequencing-based methodology for monitoring antimicrobial resistance in swine herds. J. Antimicrob. Chemother..

[14] Petersen TN (2015). Meta-genomic analysis of toilet waste from long distance flights; a step towards global surveillance of infectious diseases and antimicrobial resistance. Sci. Rep..

[15] Su JQ (2017). Metagenomics of urban sewage identifies an extensively shared antibiotic resistome in China. Microbiome.

[16] Pehrsson EC (2016). Interconnected microbiomes and resistomes in low-income human habitats. Nature.

[17] Petersen TN (2017). MGmapper: reference based mapping and taxonomy annotation of metagenomics sequence reads. PLoS ONE.

[18] Afshinnekoo E (2015). Geospatial resolution of human and bacterial diversity with city-scale metagenomics. Cell Syst..

[19] Roberts MC (2005). Update on acquired tetracycline resistance genes. FEMS Microbiol. Lett..

[20] Roberts MC (2008). Update on macrolide-lincosamide-streptogramin, ketolide, and oxazolidinone resistance genes. FEMS Microbiol. Lett..

[21] Schwarz, S., Cloeckaert, A. & Roberts, M. C. in *Antimicrobial Resistance in Bacteria of Animal Origin* (ed. Aarestrup, F. M.) 73–98 (ASM Press, Washington, D.C., 2006).

[22] Davies J, Davies D (2010). Origins and evolution of antibiotic resistance. Microbiol. Mol. Biol. Rev..

[23] van de Sande-Bruinsma N (2008). Antimicrobial drug use and resistance in Europe. Emerg. Infect. Dis..

[24] Aarestrup FM (2001). Effect of abolishment of the use of antimicrobial agents for growth promotion on occurrence of antimicrobial resistance in fecal enterococci from food animals in Denmark. Antimicrob. Agents Chemother..

[25] Dutil L (2010). Ceftiofur resistance in *Salmonella enterica* serovar Heidelberg from chicken meat and humans, Canada. Emerg. Infect. Dis..

[26] Aarestrup FM (2000). Characterization of glycopeptide-resistant *Enterococcus faecium* (GRE) from broilers and pigs in Denmark: genetic evidence that persistence of GRE in pig herds is associated with coselection by resistance to macrolides. J. Clin. Microbiol..

[27] Holmes AH (2016). Understanding the mechanisms and drivers of antimicrobial resistance. Lancet.

[28] Collignon P, Powers JH, Chiller TM, Aidara-Kane A, Aarestrup FM (2009). World Health Organization ranking of antimicrobials according to their importance in human medicine: A critical step for developing risk management strategies for the use of antimicrobials in food production animals. Clin. Infect. Dis..

[29] Martínez JL, Coque TM, Baquero F (2015). What is a resistance gene? Ranking risk in resistomes. Nat. Rev. Microbiol..

[30] Klein, E. Y. et al. Global increase and geographic convergence in antibiotic consumption between 2000 and 2015. *Proc. Natl. Acad. Sci. USA* **115**, E3463–E3470 (2018). 10.1073/pnas.1717295115 PMC5899442 29581252

[31] Vogwill T, MacLean RC (2015). The genetic basis of the fitness costs of antimicrobial resistance: a meta-analysis approach. Evol. Appl..

[32] Knudsen BE (2016). Impact of sample type and DNA isolation procedure on genomic inference of microbiome composition. mSystems.

[33] Li, H. Aligning sequence reads, clone sequences and assembly contigs with BWA-MEM. *ArXiv* https://arxiv.org/abs/1303.3997 (2013).

[34] Li H (2009). The Sequence Alignment/Map format SAMtools. Bioinformatics.

[35] Zankari E (2012). Identification of acquired antimicrobial resistance genes. J. Antimicrob. Chemother..

[36] Munk P (2018). Abundance and diversity of the fecal resistome in slaughter pigs and broilers in nine European countries. Nat. Microbiol..

[37] Ondov BD (2016). Mash: fast genome and metagenome distance estimation using MinHash. Genome Biol..

[38] Global Environment Monitoring System (GEMS). GEMS/Food consumption database (2012). (dataset: [https://extranet.who.int/sree/Reports?op=vs&path=/WHO_HQ_Reports/G7/PROD/EXT/GEMS_cluster_diets_2012]; click on “Show Data” in top-right of world-map, then “Export” in top-right of the table that has now appeared)

[39] United Nations Development Programme (UNDP). Human Development Report 2016: Human Development for Everyone (2017). (dataset: [http://hdr.undp.org/en/composite/trends])

[40] Institute for Health Metrics and Evaluation (IHME). Global Burden of Disease Study 2015 (GBD 2015) Location Hierarchies. Seattle, United States: Institute for Health Metrics and Evaluation (IHME) (2017). (dataset: [http://ghdx.healthdata.org/record/global-burden-disease-study-2015-gbd-2015-location-hierarchies])

[41] Harrison XA (2014). Using observation-level random effects to model overdispersion in count data in ecology and evolution. Peer J..

[42] Stekhoven DJ, Bühlmann P (2012). Missforest-non-parametric missing value imputation for mixed-type data. Bioinformatics.

[43] Ren Y, Zhang L, Suganthan PN (2016). Ensemble classification and regression: recent developments, applications and future directions. IEEE Comput. Intell. Mag..

[44] Zhou, Z.-H. *Ensemble Methods: Foundations and Algorithms* (Chapman and Hall/CRC, New York, 2012).

[45] Breiman L (2001). Random forests. Mach. Learn..

[46] Tibshirani R (1996). Bias, variance and prediction error for classification rules. Monogr. Soc. Res. Child Dev..

[47] Wolpert DH, Macready WG (1999). Efficient method to estimate Bagging’s generalization error. Mach. Learn..

[48] Breiman L (1996). Bagging predictors. Mach. Learn..

[49] Breiman L (1998). Bias, variance and arcing classifiers. Ann. Stat..

[50] Clark TE (2004). Can out-of-sample forecast comparisons help prevent overfitting?. J. Forecast..

[51] Kursa MB (2014). Robustness of random forest-based gene selection methods. BMC Bioinformatics.

[52] Berendsen JA (2015). The analysis of animal faeces as a tool to monitor antibiotic usage. Talanta.

[53] Bello GTJ (2016). Study of the aminoglycoside subsistence phenotype of bacteria residing in the gut of humans and zoo animals. Front. Microbiol..

[54] Berendsen JA (2013). Comprehensive analysis of ß-lactam antibiotics including penicillins, cephalosporins, and carbapenems in poultry muscle using liquid chromatography coupled to tandem mass spectrometry. Anal. Bioanal. Chem..

