# Supplementary material for: Global monitoring of antimicrobial resistance based on metagenomics analyses of urban sewage
Source: Nat Commun. 2019 Mar 8;10:1124. doi: 10.1038/s41467-019-08853-3 (PMC6408512; doi:10.1038/s41467-019-08853-3)
Supplement: Supplementary file 1 — Supplementary Information [file 41467_2019_8853_MOESM1_ESM.pdf]

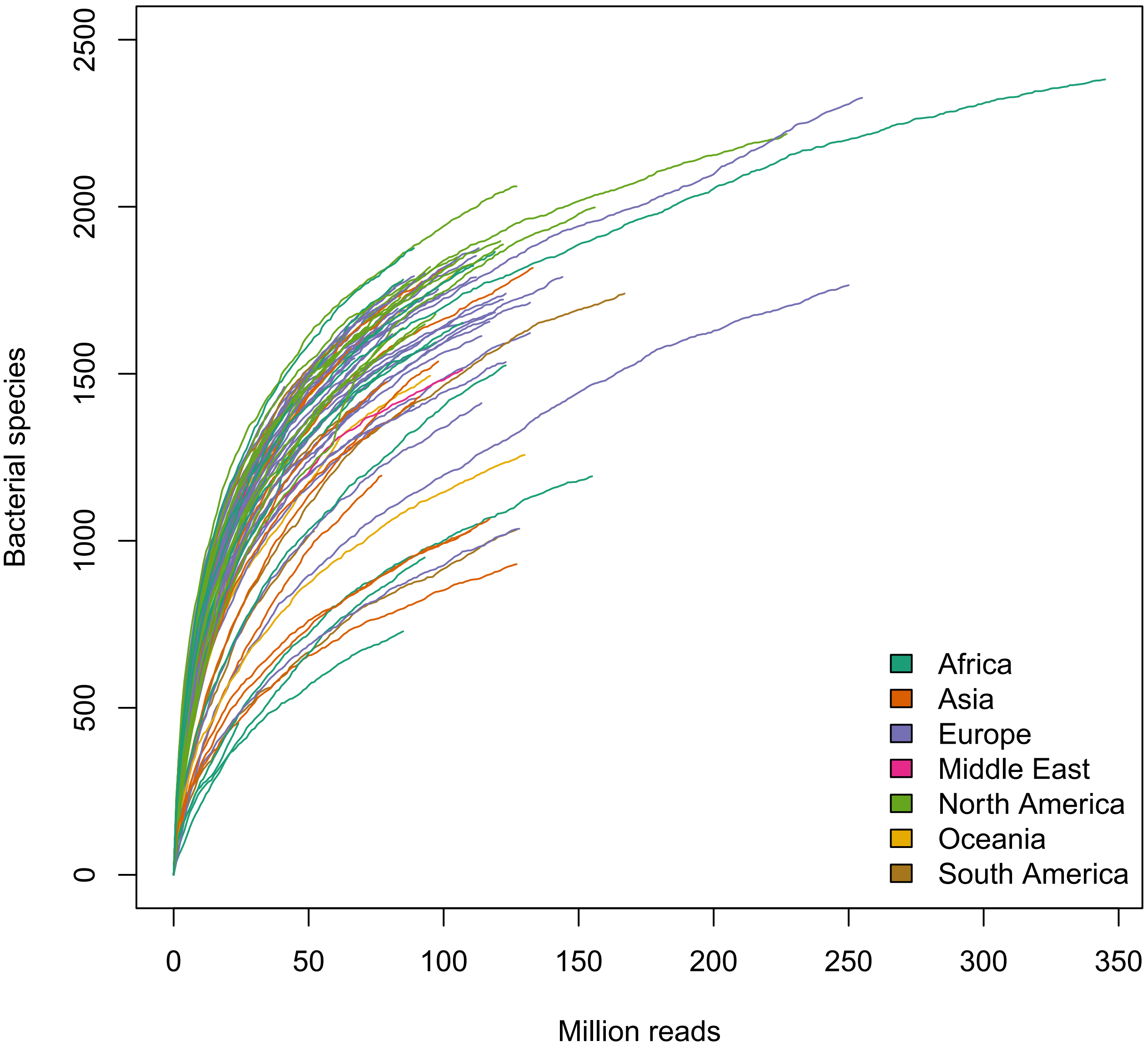

Supplementary Figure 1. Rarefaction curves for bacterial species with a minimum read count of 200.

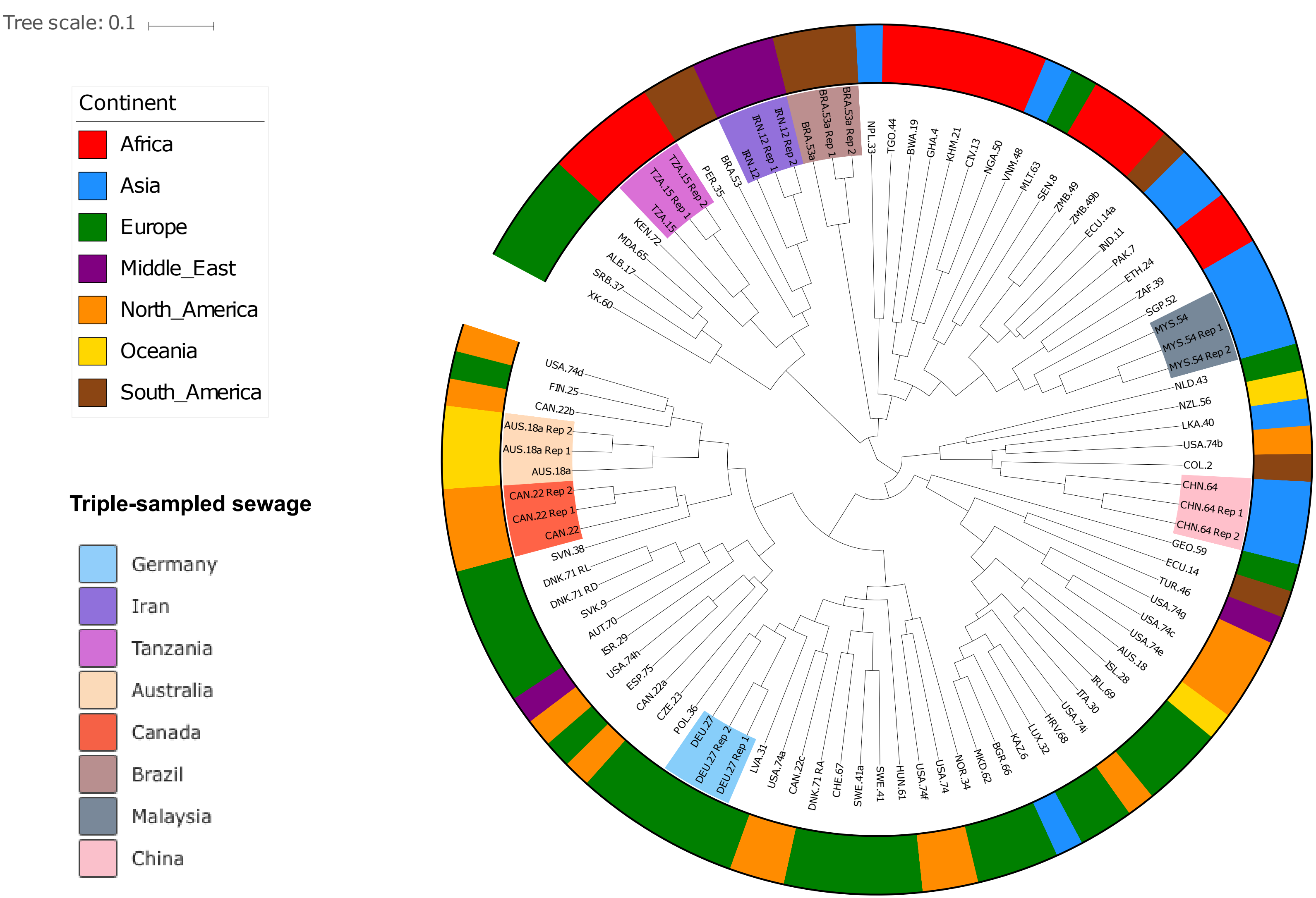

Supplementary Figure 2. Dendrogram of resistome composition from all sewage samples including double-sampled sewage from 8 countries (The colored labels inside the dendrogram). The double samples were taken one day apart. The second samples were processed twice (Rep 1 and Rep2). The day 2 samples were kept in the freezer for two years prior tom processing and sequencing. Samples from day 1 and day 2 were sequenced using the same DNA extraction and sequencing protocols.

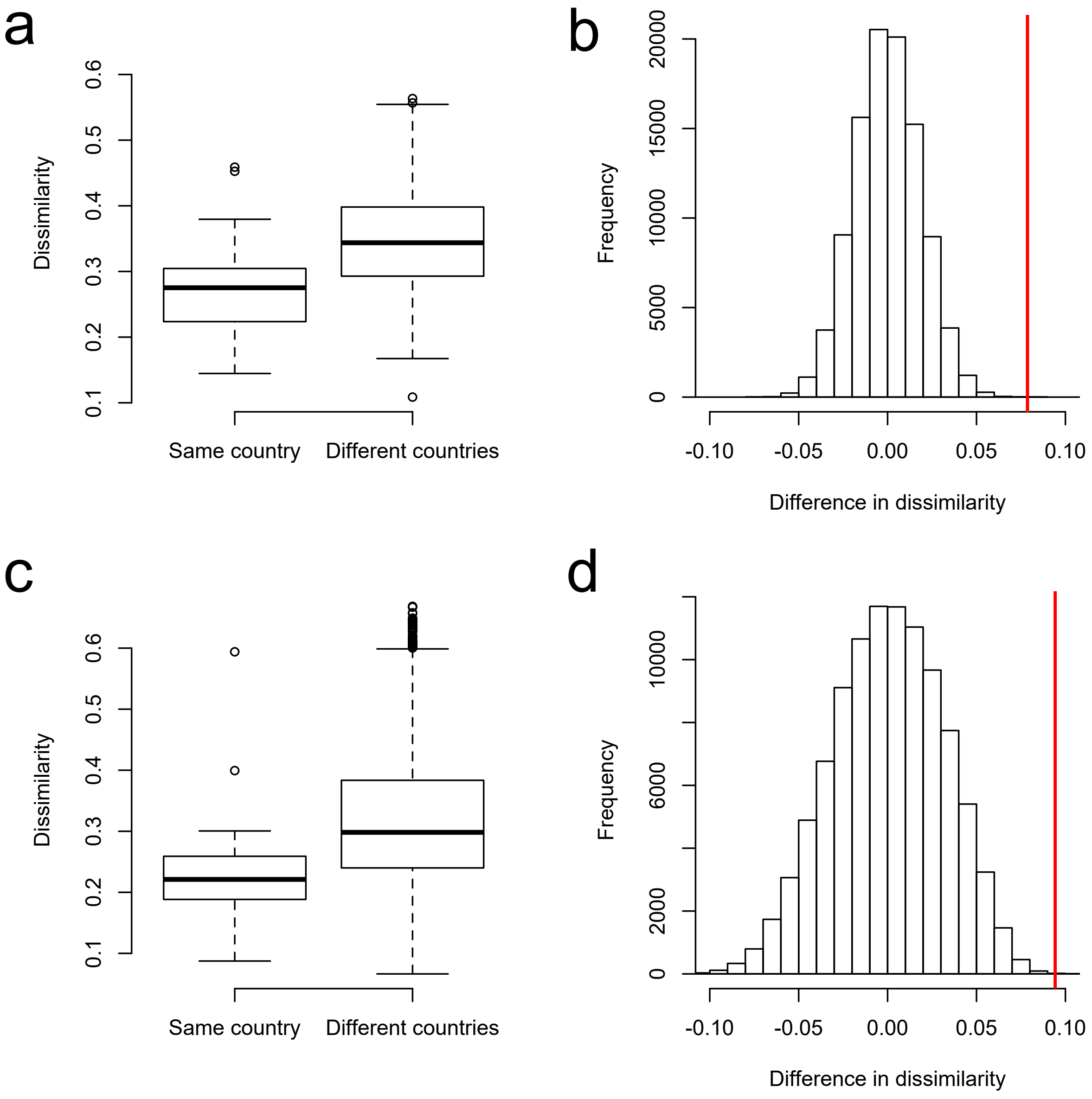

Supplementary Figure 3. Individual sites are representative of other sites in their country. (a) For the resistome the Bray-Curtis dissimilarity for pairs of sites in different countries is 34% higher than for sites in the same country. (b) Comparison of this observed difference (red line) with the null distribution from 106 permutations of the data show that this difference is significant (permutation test, $p < 0.0001$). (c) For the bacteriome the Bray-Curtis dissimilarity for pairs of sites in different countries is 46% higher than for sites in the same country. (d) Again, comparison of this observed difference (red line) with the null distribution from 106 permutations of the data show that this difference is significant (permutation test, $p < 0.0001$).

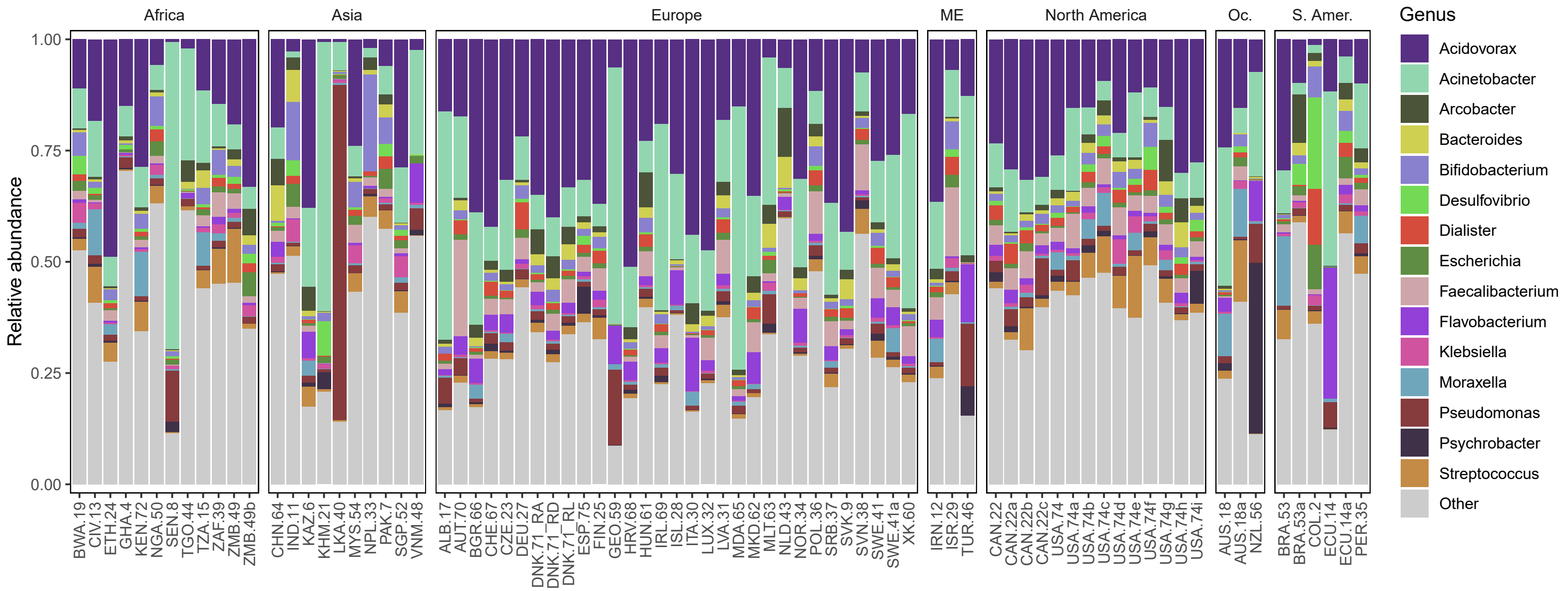

Supplementary Figure 4. Taxonomic composition of samples at genus level: the relative contributions of read pairs mapping to specific bacterial genera are shown. 'Other' captures all genera not among the 15 most abundant.

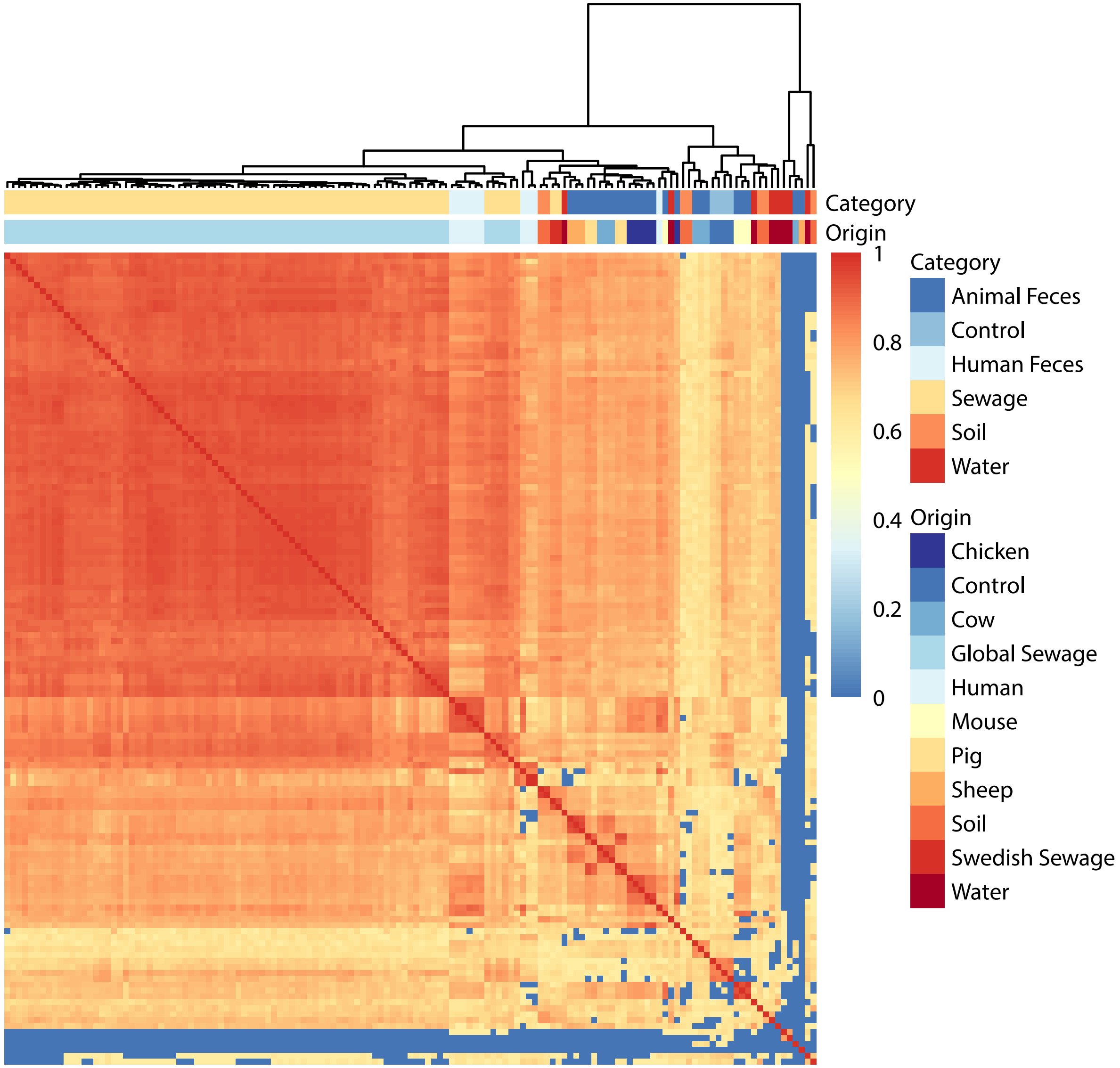

Supplementary Figure 5. Heat map of the bacterial composition of the global sewage samples in combination with available metagenomics data sets for different reservoirs and animal species. The data were generated using different DNA purification methods, but all samples were sequenced on Illumina HiSeq instruments. The global sewage samples all belong to a cluster with only human gut microbiome samples and themselves. In general, a stronger agreement was observed to human gut microbiome data compared with the other publicly available samples.

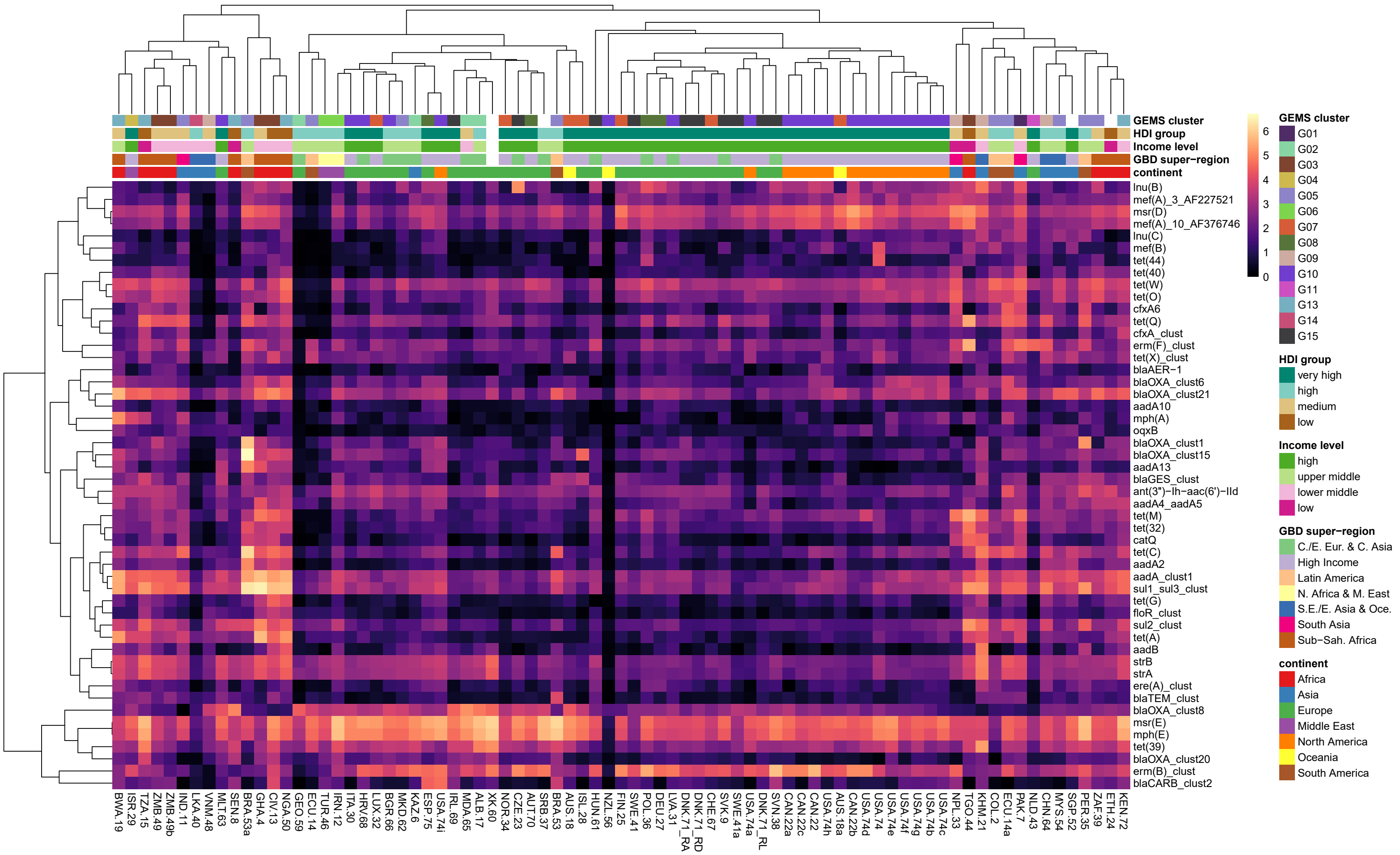

Supplementary Figure 6. AMR gene-level heat map. Relative abundances of individual AMR genes (FPKM) was combined into gene clusters with 90% sequence identity. The colors represent log (ln) transformed relative abundances (FPKM). Both gene clusters and samples were clustered using complete-linkage clustering. For genes, the clustering was done on Pearson correlation coefficients. For samples, it was done on the Bray-Curtis dissimilarity matrix, including all genes. Only the 50 most abundant AMR genes are shown here.

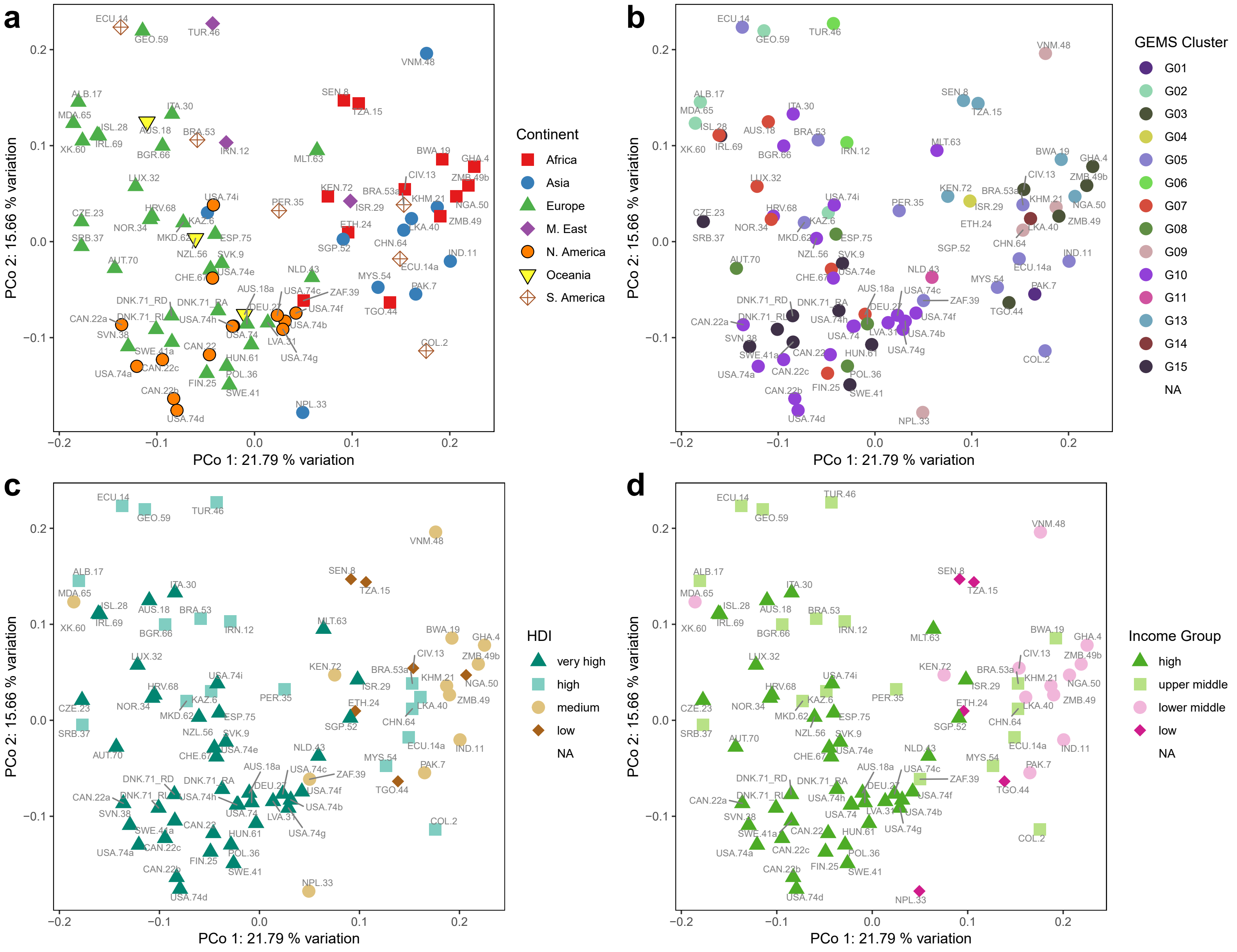

Supplementary Figure 7. Resistome sample clustering. PCoA was performed on the resistome Bray-Curtis dissimilarity matrix. The amount of variation explained by coordinates 1 and 2 is included as axes labels. Each dot labelled with country name. (a) Continents, (b) GEMS food intake clustering, (c) HDI, and (d) World Bank income.

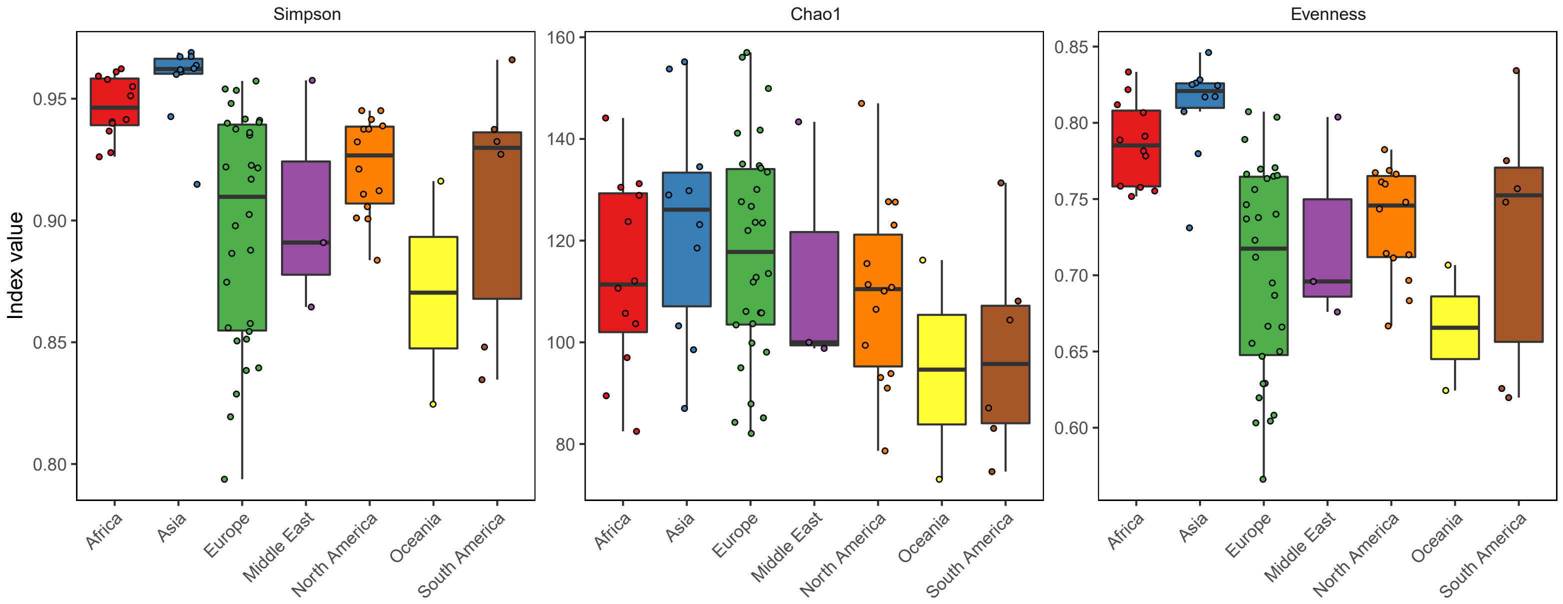

Supplementary Figure 8. Alpha diversity of AMR genes. Simpson diversity (1-D), Pielou's evenness and the Chao1 richness estimate was computed on a rarified resistance count matrix, excluding the two samples with least AMR data (<1000 hits, samples HUN.61 and NZL.56), thus subsampling to the 3rd lowest sample's level. The horizontal box lines represent the first quartile, the median and the third quartile. Whiskers denote the range of points within the first quartile – 1.5× the interquartile range and the third quartile + 1.5× the interquartile range.

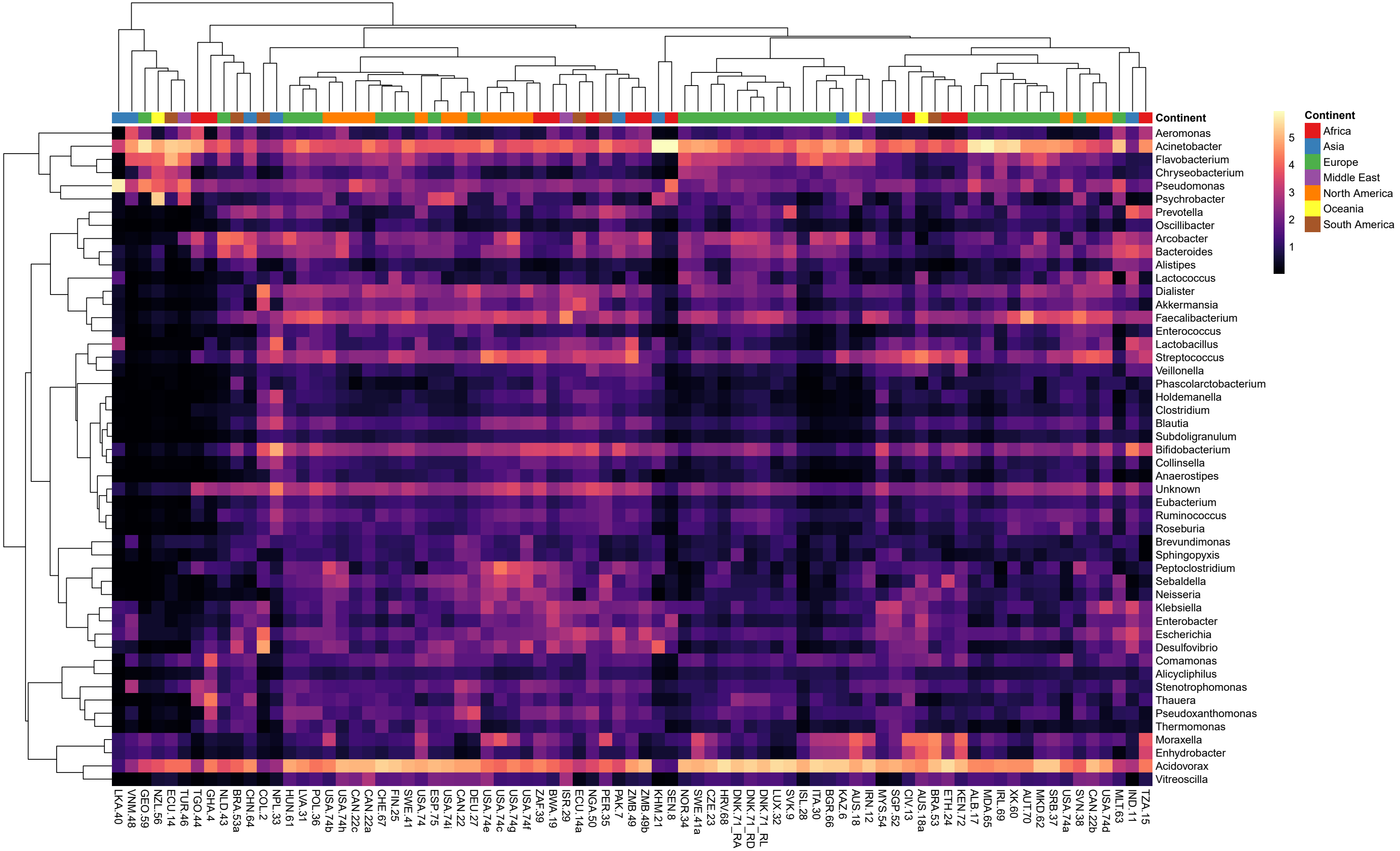

Supplementary Figure 9. Heat map of bacterial genera. Colors represent log (ln) transformed counts of read pairs. Both genera and samples were clustered using complete-linkage clustering. For genera, the clustering was done on Pearson correlation coefficients. For samples, it was done on the Bray-Curtis dissimilarity matrix, including all genera. Only the 50 most abundant genera are shown here.

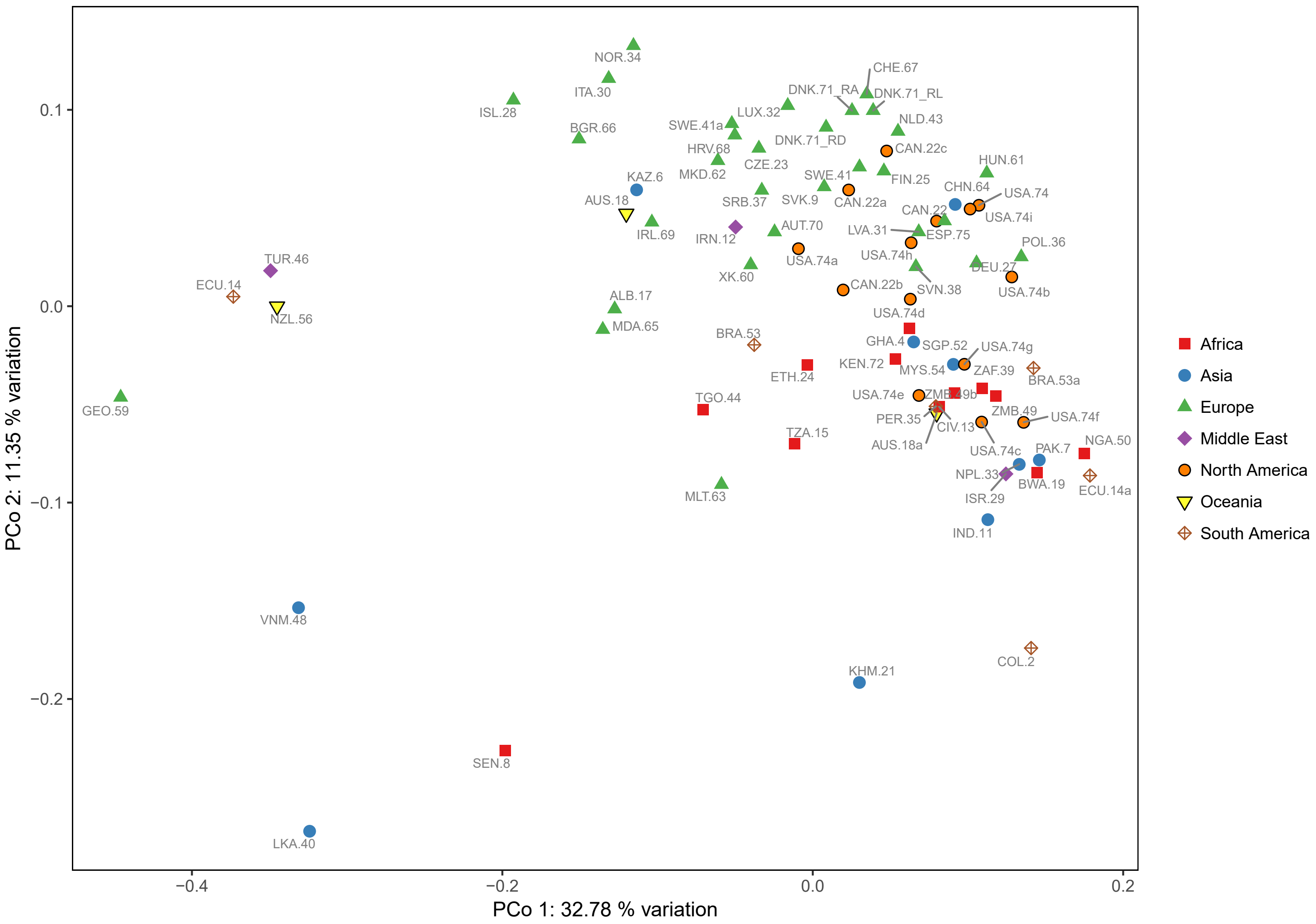

Supplementary Figure 10. Bacteriome sample clustering. PCoA was performed on the bacterial Bray-Curtis dissimilarity matrix. The amount of variation explained by coordinates 1 and 2 is included as axes labels. Geographical group explained 16.46% of the dissimilarity between sample bacteriomes (adonis2 test, $p < 0.001$).

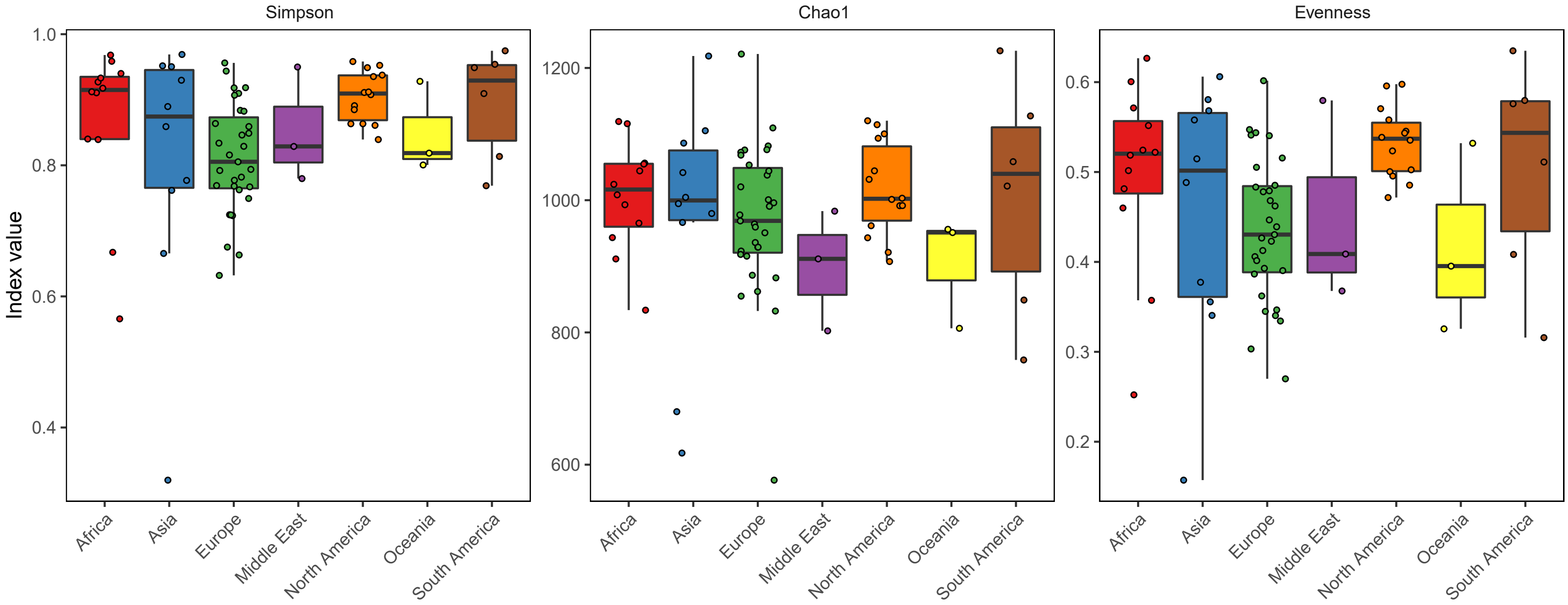

Supplementary Figure 11. Alpha diversity of bacterial genera. Simpson diversity (1-D), Pielou's evenness and the Chao1 richness estimate was computed on a rarified genus count matrix, subsampling to the sample with the least counts (>500.000).

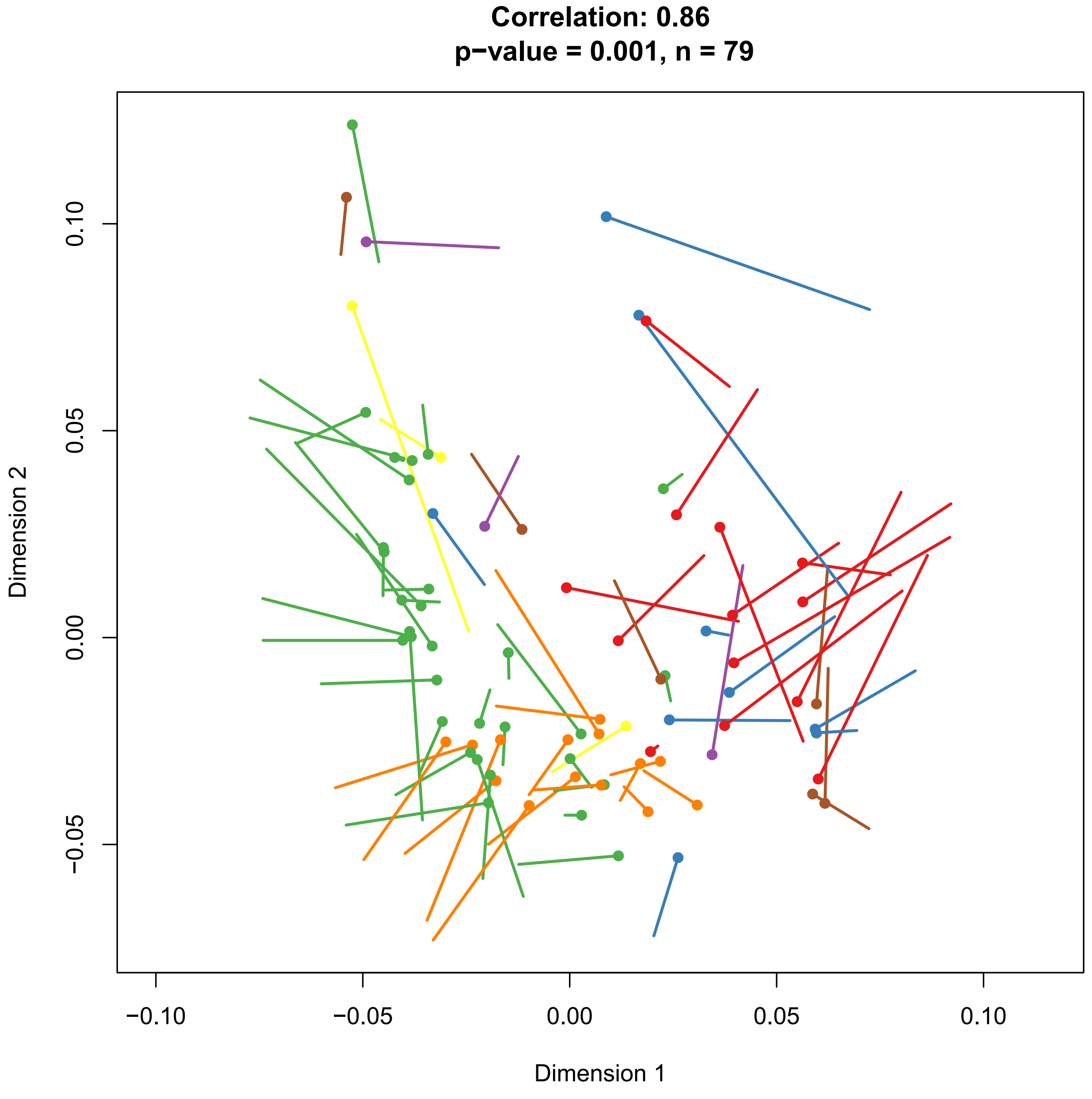

Supplementary Figure 12. Procrustes analysis was performed on the two ordinations (bacterial genera and AMR genes). The ddotted end of lines represent the resistome position, while the undotted ends represent the bacteriome position. Vegan Procrustes test 'protest' yielded a matrix-matrix correlation coefficient of 0.859 (protest, p<0.001).

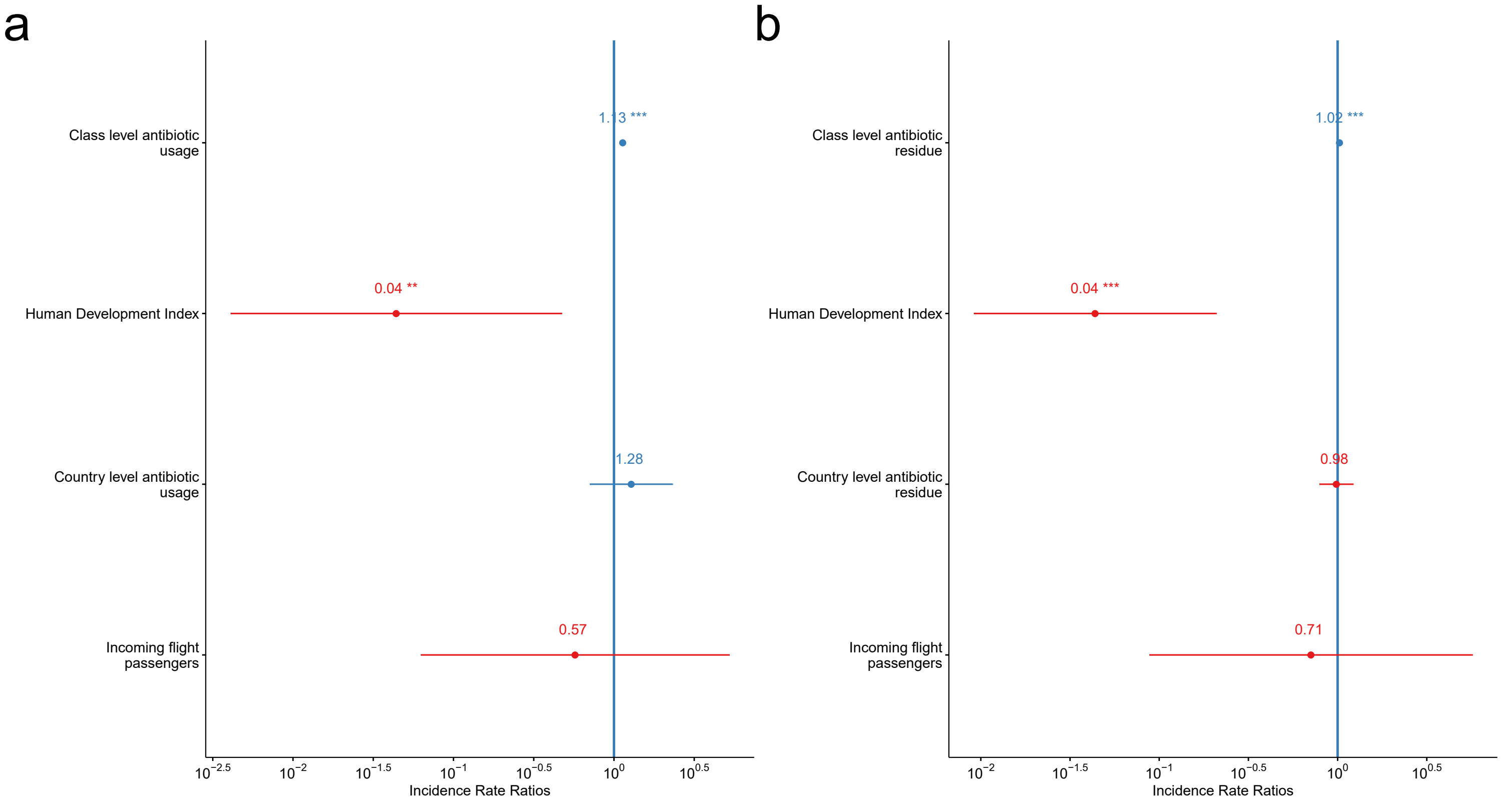

Supplementary Figure 13. Results of the generalized linear mixed effects model. (a) effects of AMU on the abundance of resistance genes, controlling for cross-resistance effects by including country wide usage and the effects of the rank in the Human Development Index and the effect of number of passengers arriving by air in a country. (b) the effects of levels of drug residue on the abundance of resistance genes, controlling for cross-resistance effects by including country wide drug residue levels and the effects of the rank in the Human Development Index and the effect of number of passengers arriving by air in a country.

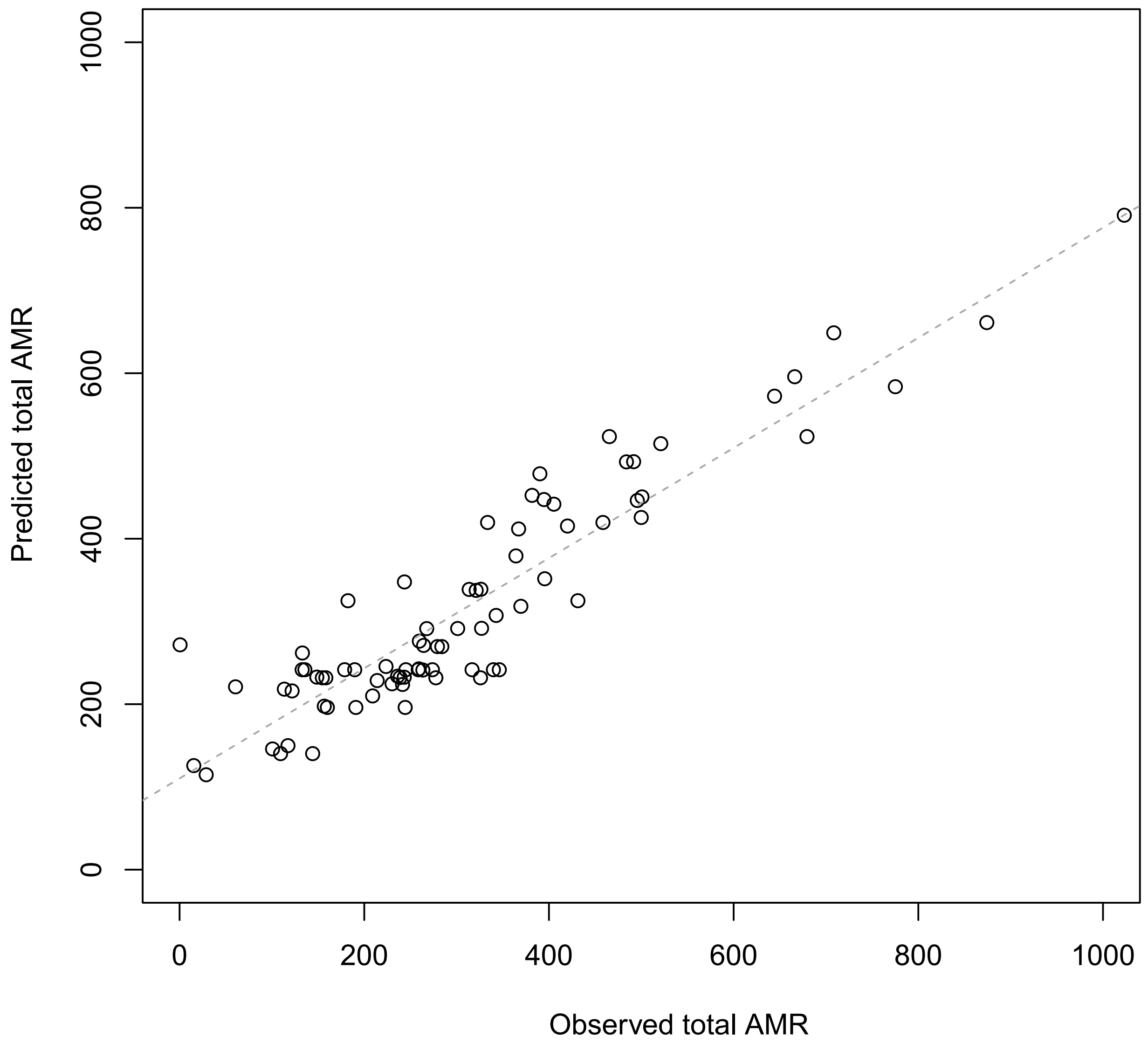

Supplementary Figure 14. Observed versus predicted FPKM values for total AMR. Global resistance predictions for the 259 countries and territories are shown in Table S9.

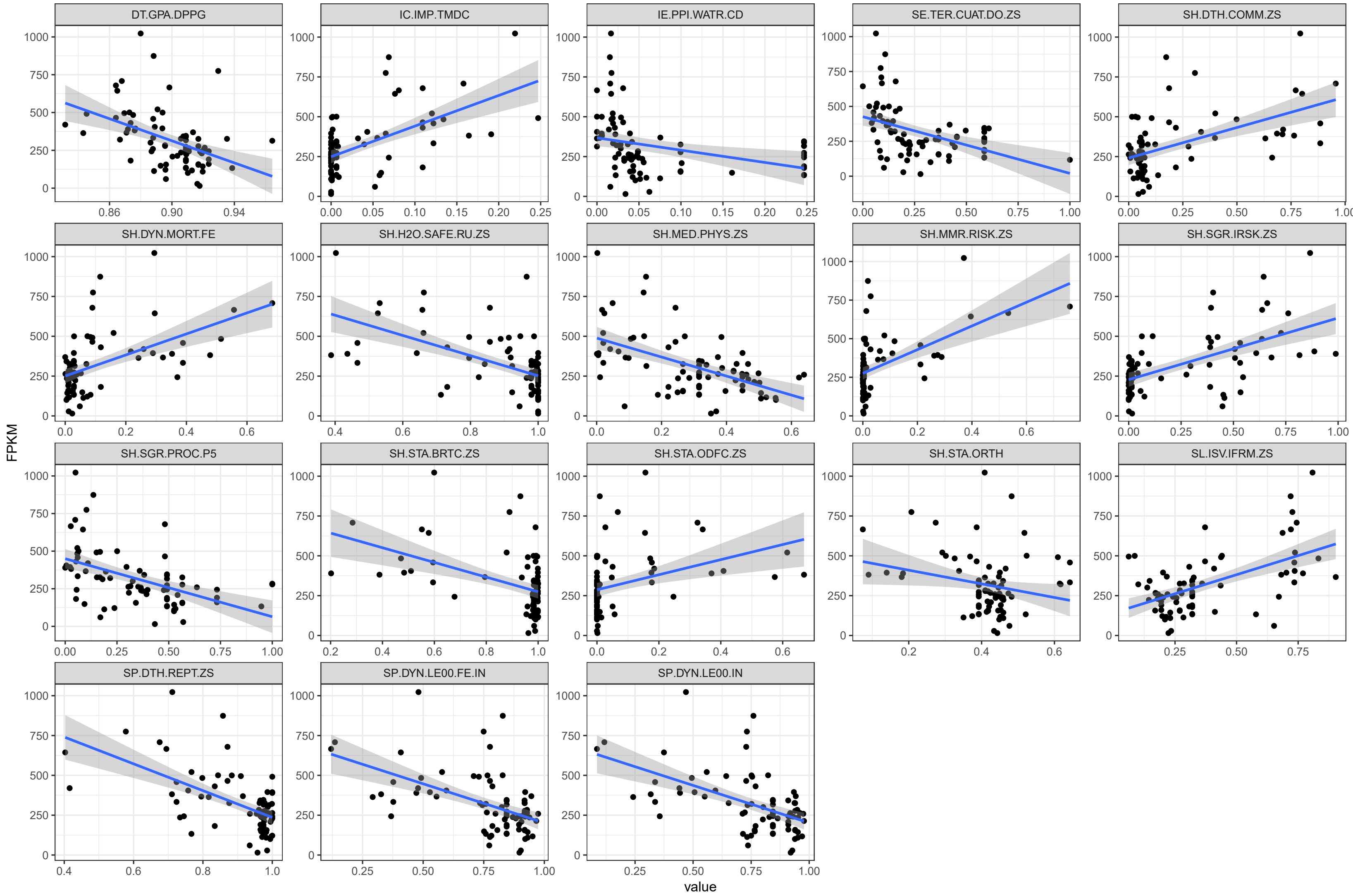

Supplementary Figure 15. Association of most important predictors with total AMR. Variable Codes: SH.DYN.MORT.FE -mortality rate, under-5, female (per 1,000 live births); IE.PPI.WATR.CD- investment in water and sanitation with private participation (current US$); SP.DTH.REPT.ZS- completeness of total death reporting (% of reported total deaths to estimated total deaths); SL.ISV.IFRM.ZS- informal employment (% of total non-agricultural employment); IC.IMP.TMDC- time to import, documentary compliance (hours); DT.GPA.DPPG- average grace period on new external debt commitments (years); SE.TER.CUAT.DO.ZS- educational attainment, Doctoral or equivalent, population 25+, total (%) (cumulative); SH.DTH.COMM.ZS- cause of death, by communicable diseases and maternal, prenatal and nutrition conditions (% of total); SH.MMR.RISK.ZS- lifetime risk of maternal death (%); SH.H2O.SAFE.RU.ZS -improved water source, rural (% of rural population with access); SH.SGR.PROC.P5-number of surgical procedures (per 100,000 population); SP.DYN.LE00.FE.IN- life expectancy at birth, female (years); SH.MED.PHYS.ZS- physicians (per 1,000 people); SH.STA.ODFC.ZS- people practicing open defecation (% of population); SH.STA.ORTH- diarrhea treatment (% of children under 5 who received ORS packet); SP.DYN.LE00.IN- life expectancy at birth, total (years); SH.SGR.IRSK.ZS- risk of impoverishing expenditure for surgical care (% of people at risk); SH.STA.BRTC.ZS- births attended by skilled health staff (% of total).

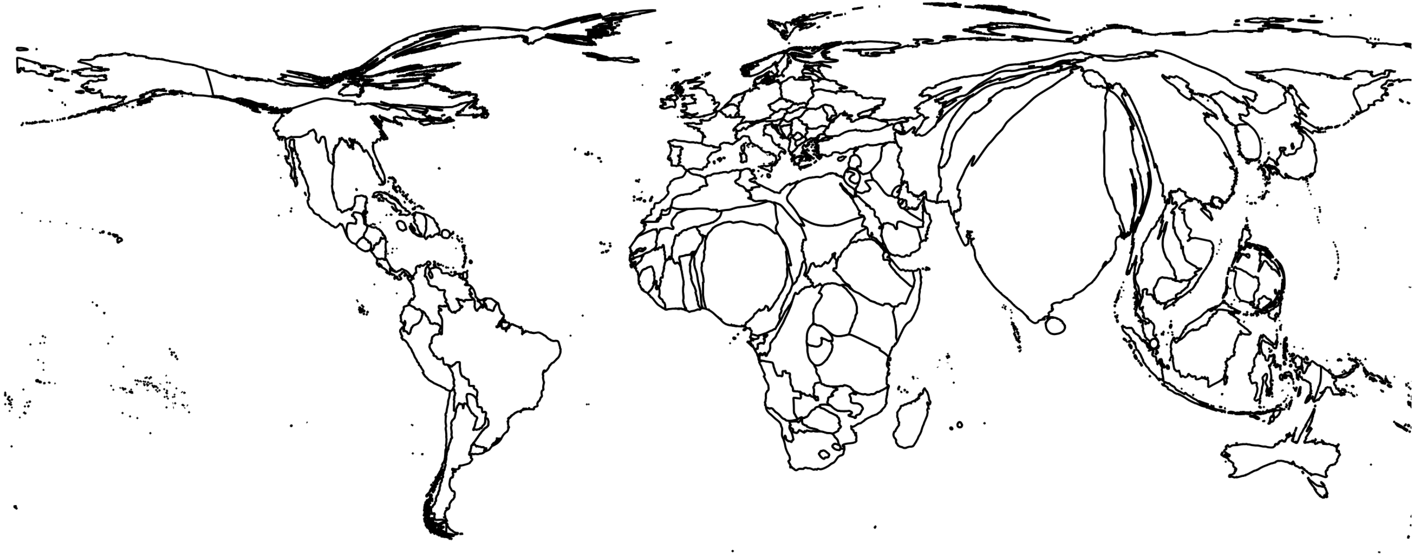

Supplementary Figure 16. AMR-distorted world map. Distortion done according to population size and predicted prevalence of AMR. The distorted map was created using the cartogram plugin in QGIS 2.18.11.

Supplementary Table 1. Results of the generalized linear mixed effects model.

	Antimicrobial usage model			Antimicrobial residues model		
	IRR	CI	p	IRR	CI	p
**Fixed effects**						
Intercept	61.44	0.06 – 62028.73	.243	388.06	1.25 – 120148.68	.042
Antimicrobial usage per am.-class	1.13	1.13 – 1.14	**<0.001**			
Country wide antimicrobial usage	1.28	0.71 – 2.32	0.414			
Passengers arriving by air	0.57	0.06 – 5.23	0.621	0.71	0.09 – 5.70	0.746
Human Development Index	0.04	0.00 – 0.47	**.0010**	0.04	0.01 – 0.21	**<0.001**
Antimicrobial residue levels per am.-class				1.02	1.02 – 1.03	**<0.001**
Country wide antimicrobial residue levels				0.98	0.79 – 1.22	0.884

Supplementary Table 2. Comparison between predicted and observed total resistance using randomForest and Support Vector Machine

Data	R^{2a}	RMSE[b]	Pearson correlation	Spearman rank correlation
RandomForest	0.87	75.1	0.93	0.87
SVM[c]	0.63	155.01	0.79	0.77

[a]Coefficient of determination (proportion of total variation of outcomes explained by the model); [b]Root mean square error; [c]Support Vector Machine

Supplementary Table 3. Important World Bank Health, Nutrition and Population and Development indicators of total AMR.

						Correlation with Cluster Variables**		
Rank	Variable	Words Bank data code	Units	Correlation* with AMR	Variables in Cluster	1st Quartile	Average	3rd Quartile
1	Mortality rate	SH.DYN.MORT.FE	under-5, female per 1,000 live births	+	37	0.47	0.63	0.80
2	Investment in water and sanitation with private participation	IE.PPI.WATR.CD	current US$	-	6	0.95	0.97	0.99
3	Completeness of total death reporting	SP.DTH.REPT.ZS	percent of reported total deaths to estimated total deaths	-	5	0.80	0.81	0.83
4	Informal employment	SL.ISV.IFRM.ZS	percent of total non-agricultural employment	+	30	0.47	0.64	0.80
5	Time to import	IC.IMP.TMDC	documentary compliance (hours)	+	7	0.73	0.81	0.91
6	Average grace period on new external debt commitments	DT.GPA.DPPG	years	-	6	0.89	0.91	0.95
7	Educational attainment	SE.TER.CUAT.DO.ZS	doctoral or equivalent, population 25+, total (%) cumulative	-	3	0.37	0.56	0.84
8	Cause of death, by communicable diseases and maternal, prenatal and nutrition conditions	SH.DTH.COMM.ZS	percent of total	+	10	0.53	0.70	0.89
9	Lifetime risk of maternal death	SH.MMR.RISK.ZS	percent	+	6	0.91	0.93	0.96
10	Improved water source, rural	SH.H2O.SAFE.RU.ZS	Percent of rural population with access	-	6	0.41	0.60	0.87
11	Number of surgical procedures	SH.SGR.PROC.P5	per 100,000 population	-	5	0.45	0.57	0.64
12	Life expectancy at birth, female	SP.DYN.LE00.FE.IN	years	-	17	0.29	0.53	0.81
13	Physicians	SH.MED.PHYS.ZS	per 1,000 people	-	11	0.41	0.64	0.85
14	People practicing open defecation	SH.STA.ODFC.ZS	percent of population	+	6	0.33	0.67	0.95
15	Diarrhea treatment	SH.STA.ORTH	percent of children under 5 who received ORS packet	+	32	0.89	0.92	0.95
16	Life expectancy at birth	SP.DYN.LE00.IN	years	-	9	0.46	0.72	0.93
17	Risk of impoverishing expenditure for surgical care	SH.SGR.IRSK.ZS	percent of people at risk	+	57	0.63	0.73	0.88
18	Births attended by skilled health staff	SH.STA.BRTC.ZS	percent of total	-	28	0.51	0.60	0.68

*Direction of association with total resistance, **Correlation between selected feature and other features from same hierarchical cluster

Supplementary Table 4. World Bank Health, Nutrition and Population as well as Development indicator variables most accurately predicting total AMR abundance using all 1,503 variables for prediction.

Rank	Variable	Words Bank Data Code	Units	Clustering with selected variable	Among the 18 variables identified through the clustering approach
1	Improved sanitation facilities, rural	SH.STA.ODFC.RU.ZS	(% of rural population with access	Selected	Y
2	People practicing open defecation, rural	SH.STA.ODFC.RU.ZS	% of rural population	Selected	Y
3	Time to import, documentary compliance	IC.IMP.TMDC	hours	Selected	Y
4	People practicing open defecation, urban	SH.STA.ODFC.UR.ZS	% of urban population	Selected	Y
5	External debt stocks	DT.DOD.DECT.GN.ZS	% of GNI	Tuberculosis case Detection rate (SH.TBS.DTEC.ZS)	N
6	People practicing open defecation	SH.STA.ODFC.ZS	% of population	Selected	Y
7	Time required to start a business, male	IC.REG.DURS.MA	days	Selected	N
8	Time required to start a business	IC.REG.DURS	days	People practicing open defecation, rural (SH.STA.ODFC.RU.ZS)	Y
9	CPIA property rights and rule-based governance rating	IQ.CPA.PROP.XQ	1 = low to 6 = high	Mortality rate under 5, female (SH.DYN.MORT.FE)	Y
10	Repeaters, primary, total	SE.PRM.REPT.ZS	% of total enrollment	Number of under five deaths (SH.DTH.MORT)	N
11	Completeness of total death reporting	SP.DTH.REPT.ZS	% of reported total deaths to estimated total deaths	Selected	Y
12	Firms that do not report all sales for tax purposes	IC.FRM.INFM.ZS	% of firms	People practicing open defecation, rural (SH.STA.ODFC.RU.ZS)	
13	Completeness of birth registration, rural	SP.REG.BRTH.RU.ZS	%	Selected	Y
14	CPIA building human resources rating	IQ.CPA.HRES.XQ	1 = low to 6 = high	Mortality rate, under 5, female (SH.DYN.MORT.FE)	Y

Supplementary Note 1

Explanation of Global Sewage sample data provided in Supplementary data 1

The sample data sheet provided as supplementary data 1 has been constructed from various data sources and is provided as a text file. Data columns have prefixes indicating their origin, allowing easy isolation / subsetting programmatically. The Rmd file shows how data was processed exactly.

In the first sheet, you will find the raw merged data. This includes lines for countries where we don't even have samples. There are multiple lines for countries with multiple samples. The 2nd sheet only has lines corresponding to the samples in the "Global Sewage" project.

First columns (no prefix): General sample-specific data from the Surveymonkey data sheet, including additional binary columns indicating whether e.g. farms or hospitals are near the sampling location, as well as GPS coordinates.

World bank data (wba): The world bank data is based on data from 2000-2016 and with imputed values in case of missing data.

Residue data (resi): This is the antimicrobial residue data. Each column states the measured concentration of a certain drug. 0-sum columns are removed, but note that many columns have very few non-0 observations.

ECDC data (ecdc): This is the human antimicrobial consumption collected from many EU countries, straitifed by drug. The data have been obtained from ECDC (www.ecdc.eu). The hospital and community usage has been summed. It is already relative to population levels (doses/people).

IMS data (ims): IMS health data from many countries. The data have been purchased under a license from IMShealth and can only be used by the consortium. The raw sales data in the dataset have been normalized to the population sizes of each country.
