# Supplementary material for: Global monitoring of antimicrobial resistance based on metagenomics analyses of urban sewage
Source: Nat Commun. 2019 Mar 8;10:1124. doi: 10.1038/s41467-019-08853-3 (PMC6408512; doi:10.1038/s41467-019-08853-3)
Supplement: Supplementary file 3 — Description of Additional Supplementary Files [file 41467_2019_8853_MOESM3_ESM.pdf]

Description of Additional Supplementary Files

File Name: Supplementary Data 1
Description: Samples and epidemiological data used for the analyses

File Name: Supplementary Data 2
Description: Read mapping results

File Name: Supplementary Data 3
Description: Bacterial genera abundances

File Name: Supplementary Data 4
Description: Antimicrobial resistance fragment per kilobase per million (FPKM) values for all sample sites

File Name: Supplementary Data 5
Description: Predicted antimicrobial, resistance levels in all countries based on selected World bank data

File Name: Supplementary Data 6
Description: Antimicrobial resistance Bray Curtis similary matriches

File Name: Supplementary Data 7
Description: World Bank variables used to predict antimicrobial resistance levels

File Name: Supplementary Data 8
Description: Protocol; Global Sewage Surveillance Project – global surveillance of infectious diseases and antimicrobial resistance from sewage
