# Supplementary material for: Global monitoring of antimicrobial resistance based on metagenomics analyses of urban sewage
Source: Nat Commun. 2019 Mar 8;10:1124. doi: 10.1038/s41467-019-08853-3 (PMC6408512; doi:10.1038/s41467-019-08853-3)
Supplement: Supplementary file 11 — Supplementary Data 8 [file 41467_2019_8853_MOESM11_ESM.pdf]

Protocol; Global Sewage Surveillance Project
# – global surveillance of infectious diseases and antimicrobial resistance from sewage

HISTORY OF CHANGES; version 2

- Added that the shipment is not IATA restricted as per SP A197 (UN labels are not required)
- Added references to Appendix 1, 2, 3, 4 and 5
- Added that an ethical approval is not required
- Paragraph on sample storage and shipment adjusted
- Editorial changes

1. EXECUTIVE SUMMARY

No single approach exists for adequately monitoring large populations and their environments for the emergence of novel pathogens. Recent developments in high-throughput sequencing offer the ability to rapidly identify nucleic acids from various organisms in clinical and environmental samples. Sewage systems are recognized as an important source of human pathogens, especially in crowded settings with poor infrastructure. A point-prevalence metagenomic analysis will be applied to sewage samples collected globally from the main sewage system of major cities prior to treatment plants inlet. The project will serve as proof-of-concept for applying metagenomic approaches, which could initiate a global surveillance of human infectious diseases including antimicrobial resistance from sewage collected in major cities around the world to detect, control, prevent and predict human infectious diseases.

2. PROJECT DESCRIPTION

Background

Human and animal populations are increasingly confronted with novel, emerging or re-emerging infectious, zoonotic, and communicable diseases including those that are multi-drug resistant. Many of these events can be attributed to increased globalization, urbanization, climate change, population growth, and intensive farming. According to the WHO, more than 25% of the total 58 million worldwide annual deaths are the direct result of infectious diseases.

Rapid detection and accurate identification of pathogens and antimicrobial resistance are essential in disease control and prevention strategies. Metagenomic analysis of genetic material through high-throughput sequencing offers the potential to greatly enhance our ability to rapidly detect emerging pathogens and related antimicrobial resistance genes. Human disease surveillance is often hampered due to ethical problems with sensitivity of data collected from individuals.

Exposure to human waste is a well-established risk factor, why sewage has been suggested as an alternative to obtain population wide samples targeting various health hazards. This could be of considerable value in attempting to establish a global human disease surveillance program because it is virtually impossible to test an entire population and obtain samples from healthy humans. To date, there have only been limited applications of metagenomic analysis for monitoring large human populations. If monitoring of pathogens and antimicrobial resistance in sewage can provide timely information on pathogens of concern, this information can be used to assist risk managers with information on appropriate prevention and treatment strategies and potential needs for environmental remediation.

This joint study between the the World Health Organization (WHO) and National Food Institute, Technical University of Denmark (DTU Food) (WHO Collaborating Centre for Antimicrobial Resistance in Food borne Pathogens) will serve as proof-of-concept for applying these metagenomic approaches to initiate a global surveillance of human infectious diseases from sewage collected in major cities around the world to detect, control, prevent and predict human infectious disease.

Description of Innovation

By applying the proposed innovative approach of using a combination of bench-top whole community sequencing (WCS) with metagenomic analysis on sewage samples collected from main sewage systems of major cities prior to treatment plants inlet, it may be possible to detect and monitor all known microbial agents and associated epidemiological markers, such a virulence and antimicrobial resistance, in a large healthy human population in relative real-time and at a low cost. This will enable the implementation of control measures that could potentially save lives and prevent further local or global spread and even predict future events.

The primary objective of the study is to evaluate the possibility of using WCS technology directly on sewage for surveillance of infectious, zoonotic, and communicable diseases in a global context. This will be achieved through the collection of two random consecutive and representative sewage samples from each city representing the entire population with connection to the main sewage outlet. Each sample will consist of approximately 1L of sewage collected at a designated sampling point (e.g. main sewage pipe prior to waste water treatment plants (WWTP inlet or disposal directly into rivers or similar, following a detailed sampling protocol (see below)). Sampling locations will be georeferenced by GPS and an image of the sampling site will be captured. The samples will be stored at –80°C until shipping to the National Food Institute, Technical University of Denmark (DTU Food) for WCS and further metagenomic analysis. The shipment is not IATA[1] restricted as per SP A197 (see Appendix 2 and 3), this means that the parcel can be sent without a UN-label.

At DTU Food, extraction of DNA and sequencing will be performed using Illumina Hiseq technology. An expected 50-100 million reads will be sequenced from each sample. A subset of collected sewage samples will be spiked with a constructed pool of known pathogens to evaluate sensitivity of the extraction and sequencing methodology.

[1] International Air Transport Association

Different available and in-house programs hosted by DTU's Center of Genomic Epidemiology will be used to filter data and map sequence reads against databases of reference genomes (ca. 2,000), selected genes, and human microbiome data (MetaHIT gene catalogue and HMP genomes). The remaining part of the sequence reads, that do not map to anything, will be compared with Genbank (NT) using Blastn. For all abundant microbial taxonomic groups, the matching sequence reads will be counted and used to define an abundance profile. The data will be analyzed and presented per country and subsequently be related to available data such as antimicrobial usage. At a later stage, additional hazards such as the content of virus and parasites as well as bacterial virulence markers will be examined. All sequence data will be submitted and deposited in the public domain at NCBI /ENA.

Impact

The most important outcome will be a proof-of-concept of "real-time" large-scale population surveillance combining state-of-the-art technology and analytic facilities that provide better and faster detection and control of health risks. In addition, the project is also expected to provide a proof-of-principle regarding the evaluation of WCS performed directly on sewage samples. The impact from this project could establish the foundation for the first surveillance of a large, healthy human population and possibly animal populations. Thus, it could reduce morbidity and mortality through rapid disease detection, reduce the development of antimicrobial resistance through proper drug adherence and enable earlier clinical treatment (interventions?), and ultimately improve treatment outcome and minimize disease spread. The outcome of this study could lead to a complete paradigm shift in the way infectious disease surveillance of nationwide or disease hot spots are conducted.

Key risks

It is impossible to accurately predict whether infectious diseases will be present in the sampling site or period. To-date, WCS studies have used clinical samples such as urine, blood and feces with success; however, there is limited knowledge on the sensitivity and specificity of WCS from sewage samples. In addition, there may be a limitation in the detection of all microbial agents since this depends on the availability of reference data. However, the knowledge gained even if the expected outcomes are not achieved will be valuable since it will identify the limitation in the proposed approach and technology enabling researchers to focus on those areas for future applications.

Ethical Issues

All analysis will be conducted in accordance with the Danish Act on scientific ethical treatment of health research administrated and confirmed by the Research Ethics Committees of the Capital Region of Denmark (www.regionh.dk), Journal no.: H-14013582. Thus, it will not be possible to trace back samples or data to any individuals, i.e. an ethical approval is not required.

Publication and IP

The samples will be stored temporarily at DTU prior to disposal. The samples will not be transferred to third parties and will solely be used for this international sewage project. All metagenomic raw data for each individual sample will be made available for the individual sample provider as soon as the results are available and subsequently deposited in the public domain at the time of publication. All metagenomic data in combination with minimum meta-data (location and date) for all samples will be made available for the COMPARE consortium (www.compare-europe.eu).

Please contact Dr. Rene S. Hendriksen rshe@food.dtu.dk from DTU Food if there is a need for signing a contract, agreement or material transfer agreement (MTA).

It is expected that the study will result in a number of scientific publications. It is expected that all partners, either country mediator or sample providers, will be co-authors on the first publication (e.g. one per country). Persons also actively participating in the analysis of the samples and/or data analysis may become co-authors on multiple publications. All co-authors will have the opportunity to comment on the manuscript prior to submission.

3. DETAILED PROCEDURE

Sampling site

The intention is to collect two samples representative of the entire population with connection to the main sewage outlet. Note that all WWTPs will most likely have an individual infrastructure and the project design allows for some individual variations as to the exact spot from where to collect the samples.

Not all sites have special equipment for continuous sampling concentrated over an entire day, and we will have to rely on crude point sampling in these cases.

From each location, two representative sewage samples (1L each, in total 2L) are collected from the main sewage flow on consecutive days from the city's main sewage pipelines prior to WWTP inlets or from the main outlet to rivers or similar. Samples can be obtained following the first filtering step, but it is important that there has been no processing of the sewage.

It is preferred to collect concentrated flow proportion sampling over 24 hours, however, should this not be possible due to lack of equipment, three crude point samples should be collected in a short time interval, i.e. at least 5 minutes between each individual sample, to ensure as much randomness as possible.

An image of the sampling site should be captured indicating the sampling environment. Please send the image to Mrs. Susanne Carlsson (suca@food.dtu.dk) indicating in the file name country and city. Please also share epidemiological data for each sample, including GPS coordinates of sampling location; WGS84 geodetic datum (e.g. N43°38'19.39'' / W116°14'28.86'') also used by Google Earth, temperature of the sample at the time of sampling, storage temperature of sample, etc.

A survey has been set up to collect these data, as well as other general information. Please submit a.s.a.p. after sampling via the following SurveyMonkey link: https://www.surveymonkey.com/r/SewageSampleDetails (see also Appendix 5)

Sampling procedure

A checklist relevant for the sampling is listed in Appendix 1.

Please follow the below basic instructions for each sample:

1) Using a permanent marker, label two clean 1000 mL plastic containers (no soap or disinfectant residue) marked with country, city, name of the collector, sample number (001 or 002) and date. Seal the label with tape to avoid the text being smeared.
2) Fill 1L of sewage into each of the containers – one per day. Leave a bit of space to allow the sewage to expand during freezing.
    a) Collect each flow proportion sewage sample over 24 hours if possible from the mid-stream of the sewage inlet to the WWTP.
    b) In the case where it is not possible to collect samples over a 24-hour period e.g. the sewage is not treated and runs directly into rivers, then collect the sample in short time intervals, i.e. with at least 5 minutes between each of three individual sample of approximately 300 ml, and subsequently pool the three samples to ensure as much randomness as possible.
    c) Record the temperature of the sewage flow the sample is taken from.
3) Close the containers with the corresponding cap and tighten to avoid leakage. The sample must be kept as cool as possible and transported to the local laboratory within 8 hours of collection. Wipe the surface of the containers clean with alcohol. Pack each container in a plastic bag individually storing at -80°C as soon as possible.

Sample storage and shipment

Store the containers at -80°C for at least 48 hours (preferred) and prepare shipping the samples to the DTU Food in Denmark. The samples should be packed according to the description in Appendix 2. See also Appendix 4 that presents an example of the packing procedure. The samples should not be shipped using dry ice as this will complicate shipping and increase costs, for the same reasons, the shipment should be sent without temperature restrictions (no cool-chain necessary during shipment). Please send the samples frozen, packed directly from the freezer.

The international courier services of DHL or FedEx must be used. Please use the shipping account numbers provided below:

- DHL: 951 717 051
- Fedex: 468 724 260

The full shipping address is:

National Food Institute, Technical University of Denmark
Søltofts Plads, Building 221, 2nd floor, room 208
2800 Kgs. Lyngby
DENMARK
Att.: Susanne Carlsson
Phone: +45 35 88 66 10
suca@food.dtu.dk

Timeline

The overview of the project will be as indicated in the table below.

The sampling window in which all participants should arrange for the sampling to take place will be the last week of January and first week of February.

**Activity**	**1 (December '15)**	**2 (January '16)**	**3 (February '16)**	**4 (March '16)**	**5 (April '16)**	**6 (May '16)**	**7 (June '16)**	**8 (July '16)**	**9 (August '16)**	**10 (September '16)**	**11 (October '16)**	**12 (November '16)**
Sign agreements / MTAs	x	x										
Collect sewage samples		Last week	First week									
Ship sewage samples			x									
DNA / RNA extraction				x								
Whole Community Sequencing					x	x						
Bioinformatics analysis							x	x	x	x	x	
Final report												x

Proposed Funding Source

This project is jointly funded by DTU Food and WHO and supported by the European Union's Horizon 2020 research and innovation programme under grant agreement No. 643476, COMPARE (www.compare-europe.eu).

Specific Safety Requirements and Responsibilities

This protocol describes how to collect sewage samples containing human and potentially animal faeces. Blood- and airborne pathogen protection (relevant personal protection: gloves, lab coat, mask, etc.) must be utilized when handling human and animal clinical samples.

Biosafety level-3 (BSL-3 / RG-3) practices and procedures must be followed when handling sewage or clinical samples suspected to contain pathogenic organisms. It is the responsibility of the collector and local laboratory to comply with the biosafety rules.

--- --- ---

PROTOCOL for Global Sewage Surveillance Project - APPENDICES

Appendix 1: Checklist when sampling

Appendix 2: Packing the sewage samples

Appendix 3: Contents of shipment (letter)

Appendix 4: Packing the sewage samples – EXAMPLE

Appendix 5: Overview of Internet-based survey

Appendix 1
Checklist when sampling

Remember to bring:

- 2 x sample containers
- A permanent marker (water proof pen)
- Tape
- Thermometer for measuring the temperature of the sample
- Camera or smartphone for taking a photo
- GPS or smartphone for taking note of the GPS-coordinates
- A print of Appendix 5 (the list of questions to respond to in the survey)

For Appendix 5, remember to take note of:

- The date and time of sampling
- The temperature of the day of sampling and the day before sampling
- The weather of the day of sampling and the day before sampling
- The transportation time from sample site to storage
- The transportation temperature from sample site to storage
- The storage temperature until shipping

Use the sampling procedure as indicated in the protocol:

1) Using a permanent marker, label two clean 1000-ml plastic containers (no soap or disinfectant residue) marked with
    - country
    - city
    - name of the collector
    - sample number (001 or 002) and
    - date

   Seal the label with tape to avoid the text being smeared.

2) Fill 1L of sewage into each of the containers – one per day. Leave a bit of space to allow the sewage to expand during freezing.
   a) Collect each flow proportion sewage sample over 24 hours if possible from the mid-stream of the sewage inlet to the WWTP.
   b) In the case where it is not possible to collect samples over a 24-hour period e.g. the sewage is not treated and runs directly into rivers, then collect the sample in short time intervals, i.e. with at least 5 minutes between each of three individual sample of approximately 300 ml, and subsequently pool the three samples to ensure as much randomness as possible.
   c) Record the temperature of the sewage flow the sample is taken from.
3) Close the containers with the corresponding cap and tighten to avoid leakage. The sample must be kept as cool as possible and transported to the local laboratory within 8 hours of collection. Wipe the surface of the containers clean with alcohol. Pack each container in a plastic bag individually storing at -80°C as soon as possible.

--- --- ---

Appendix 2

Packing the sewage samples

The shipping of the sewage samples to Denmark is not IATA[1] restricted as per SP A197.

(Note: the contents of the parcel fall under the UN-category *UN3082 environmentally hazardous substance, liquid, n.o.s.* – **BUT** since the amount shipped is less than **5L**, the consequence is that it is **not IATA restricted as per SP A197**)

This means that when shipping the parcel, there should be no UN-diamond label on the outside of the parcel.

At departure, the sample material must be frozen (at -80°C), and the parcel must be packed to keep the sample material frozen for as long as possible, must protect the contents from leaking, and it must ensure that the liquid is collected inside the parcel, should one of the sample containers leak. This is described as follows by IATA in A197 and references (5.0.2.4.1, 5.0.2.6.1.1 and 5.0.2.8).

IATA A197 and references indicate that the packaging used must be good quality packagings which must be strong enough to withstand the shocks and loadings normally encountered in transport, including removal from a pallet, unit load device or overpack for subsequent manual or mechanical handling. Packages must be constructed and closed as to prevent any loss of contents when prepared for transport which might be caused under normal conditions of transport, by vibration or by changes in temperature, humidity or pressure (resulting from altitude, for example). Packages (including inner packagings and receptacles) must be closed in accordance with the information provided by the manufacturer. No dangerous residue must adhere to the outside of packages during transport. These provisions apply, as appropriate, to new, reconditioned or remanufactured packagings.

Parts of packagings which are in direct contact with the sample material:

a) Must not be affected or significantly weakened by the sample material;
b) Must not cause a dangerous effect, e.g. catalyzing a reaction or reacting with the sampling material; and
c) Must not allow permeation of the sample material that could constitute a danger under normal conditions of transport

When filling packagings for liquids, sufficient ullage (outage) must be left to ensure that neither leakage nor permanent distortion of the packaging will occur as a result of an expansion of the

[1] International Air Transport Association

liquid caused by temperatures likely to prevail during transport. Liquids must not completely fill a packaging at a temperature of 55°C.

In summary:

When shipping, the sewage samples must be frozen at -80°C.

The contents of one parcel must not exceed 5L of waste water.

Packagings must be strong and of good quality.

The primary receptacles should be packed in secondary packagings in such a way that, under normal conditions of transport, they cannot break, be punctured or leak their contents into the secondary packaging. Secondary packagings should be secured in outer packagings with suitable cushioning material. ***Any leakage*** *of the contents must not compromise the integrity of the cushioning material or of the outer packaging*.

Any instructions from packaging manufacturers or distributors on filling and closing the packages must be followed to enable the package to be correctly prepared for transport.

On the external surface of the outer packaging

One label is necessary; i.e. 'This way up' indicated by two arrows (if it is not already printed on the external surface of the outer packaging). This example of the looks of the label may be printed and taped to the parcel.

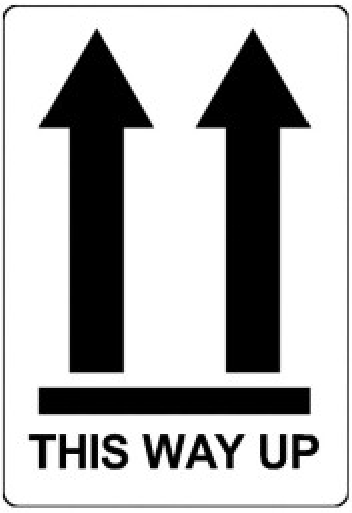

No diamond-shaped labels are required for the shipment of this parcel (because the content is not IATA restricted as per SP A197).

Indicate the name and address of the shipper and of the consignee.

Documentation

In the AWB for the ‘full desription of contents’, indicate **Watersamples, not IATA restricted as per SP A197**.

In Appendix 3 you find a letter from Professor Frank Aarestrup. This indicates the contents of the shipment, and also states that the shipment does not contain dry-ice.
Print the letter, and make sure to add it with the AWB when sending the parcel.
(the letter is intended for those that handle the parcel that when they see on the x-ray that something in the parcel is very cold, they are informed that it is not a sign of dry-ice, and to confirm to them that the parcel is not IATA restricted as per SP A197)

An import permit is <u>not</u> required.

A Shipper's Declaration for Dangerous Goods is <u>not</u> required.

--- --- ---

National Food Institute

DTU

To whom it may concern

Kgs. Lyngby 5 January 2016
/suska

Contents of shipment

This shipment contains samples of waste water (no more than 5L) for the purpose of laboratory analysis.

At departure, the sample material was frozen (at -80°C).

This shipment does not contain dry ice.

The shipment is not IATA restricted as per SP A197.

Kind regards,

Frank M. Aarestrup
Professor

Technical University of Denmark
National Food Institute

Søltofts Plads
Building 221
2800 Kgs. Lyngby
Denmark

Tel +45 3588 6601

suska@food.dtu.dk
www.food.dtu.dk

Appendix 4

Packing the sewage samples - example

This appendix presents **an example** for packing the sewage samples. When shipping the sewage samples to Denmark, the samples must be packed according to Appendix 2 in the Global Sewage Surveillance protocol.

1 (packing example)

Sewage sample is filled into sample containers

Make sure to leave empty space in the sample containers

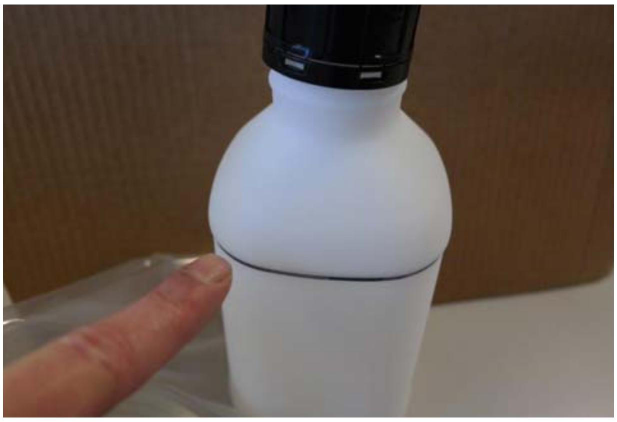

2 (packing example)

After filling sewage sample into the sample container, close them firmly

Wipe each sample container off with ethanol or another disinfectant

Mark each sample container with sample information as indicated in the protocol (sampling procedure)

Place each sample container in a plastic bag

Close the plastic bag e.g. using zip

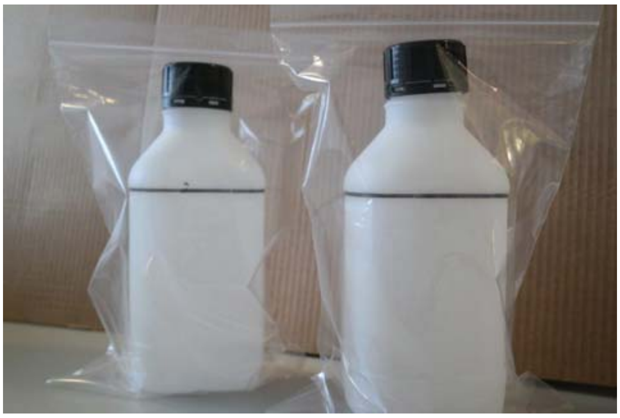

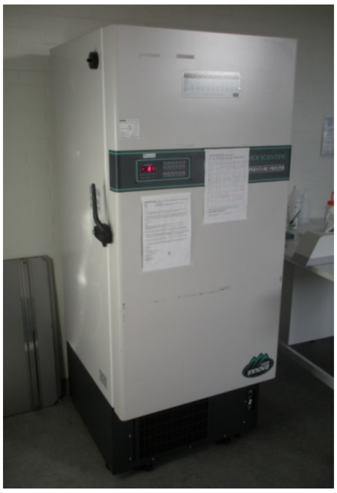

3 (packing example)

Place the sample containers in the -80 ^{o}C freezer at least 48 hours until completely frozen

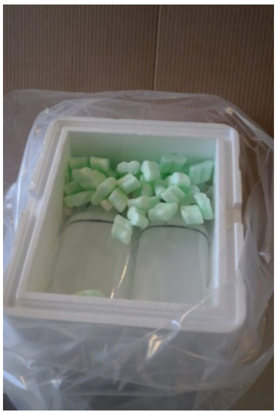

4 (packing example)

Place the polystyrene box in a large plastic bag

Place the sample containers containing the frozen sewage samples in a polystyrene box

Cover with polystyrene stuffing

5 (packing example)

Cover the frozen sewage samples with vermiculite

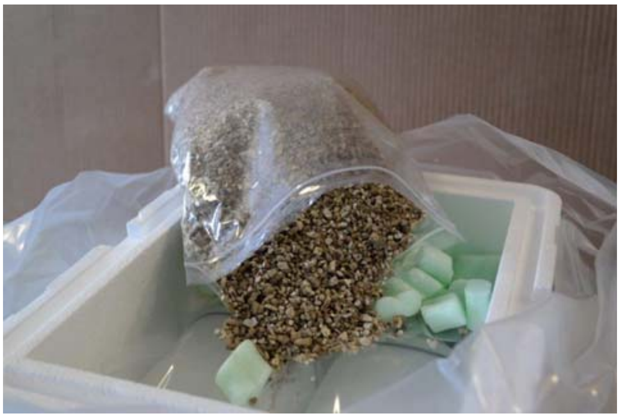

6 (packing example)

Make sure the box is full, meaning that

- The samples must not move inside the box when 'shaking' the package

- The box must not be too full, but must be easily closed with the lid

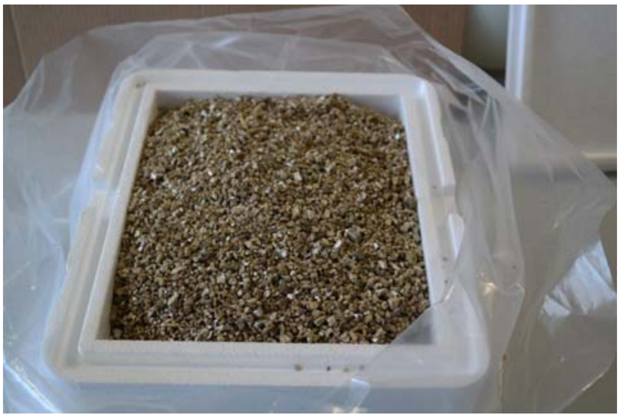

7 (packing example)

Place the lid on the box

Use tape (could be fortified tape) for fixing the lid thoroughly to the box, i.e. all the way around the box where the lid is joined to the box

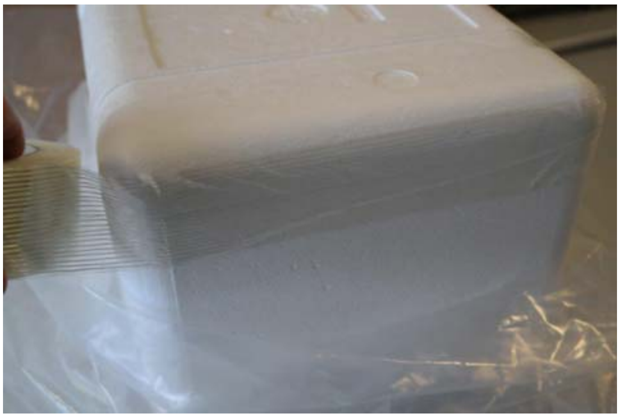

8 (packing example)

Make sure the tape fixes to the box

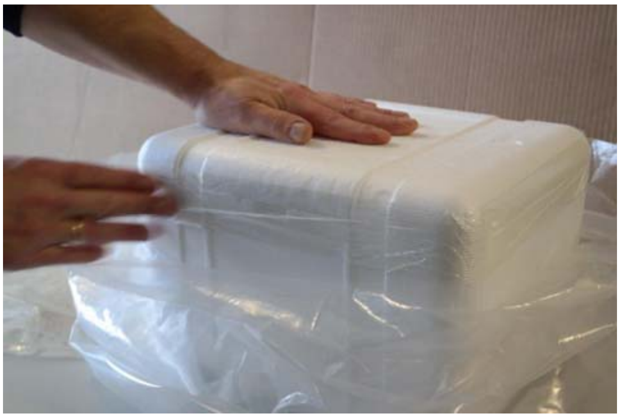

9 (packing example)

Fold the plastic bag and close it using tape

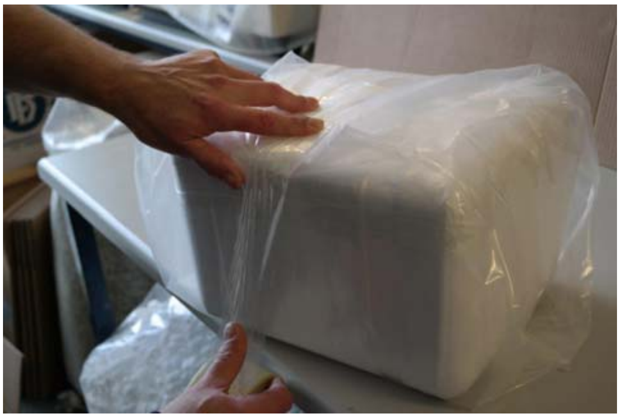

10 (packing example)

Make sure the tape is fixed well down the sides of the box

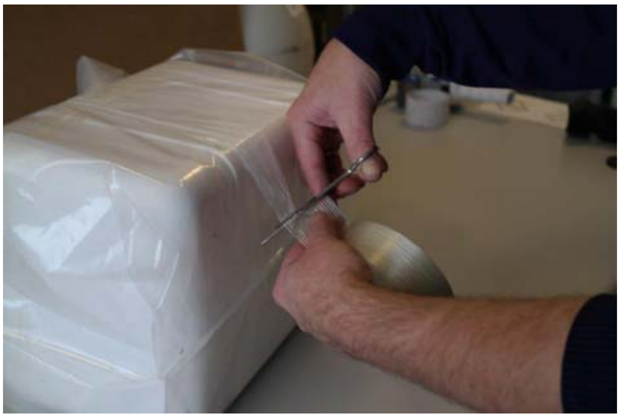

11 (packing example)

With the palm of your hand, press gently on the tape allowing it to fix well to the plastic bag

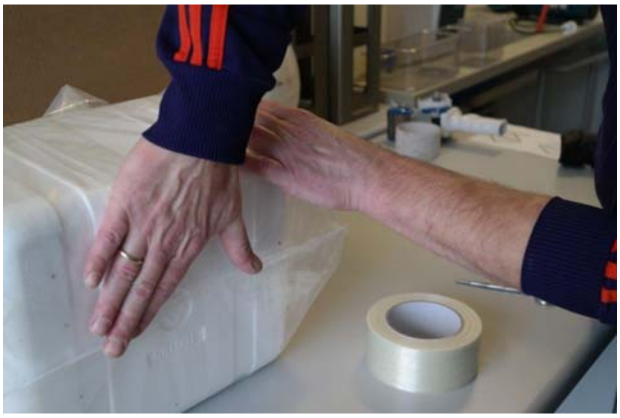

12 (packing example)

Place the closed bag containing the polystyrene box inside the cardboard box

Make sure the polystyrene box does not move inside the cardboard box when ‘shaking’ the package

Make sure to tape all way around the parcel three times, i.e. once in the middle fixing the flaps, and twice all the way around the sides of the parcel as indicated on the photo

Make sure the ends of the tape fix each other (see photo)

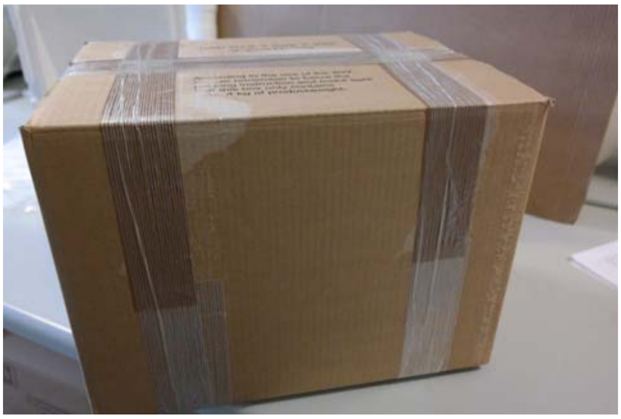

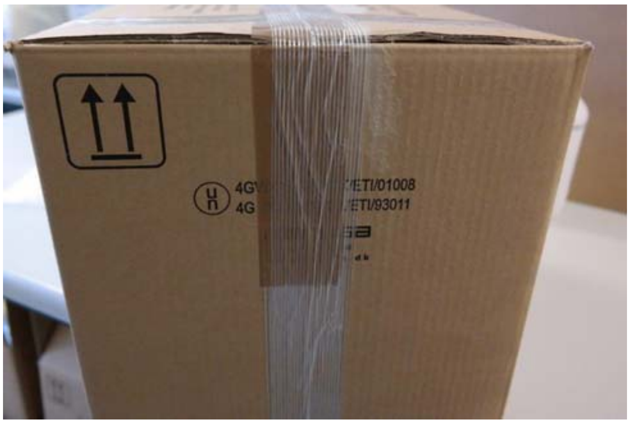

13 (packing example)

Make sure the tape correctly fixes the flaps to the cardboard box

14 (packing example)

Make sure that all corners and ends of the fortified tape is fixed to the cardboard box

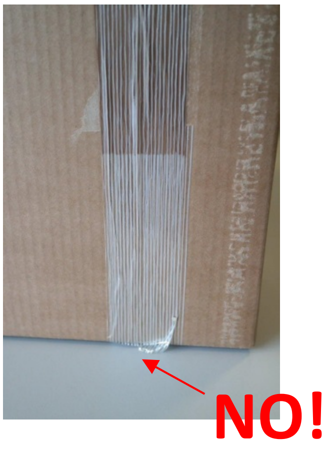

15 (packing example)

This photo indicates how 'an open corner of tape' can be fixed over itself or behind another piece of tape

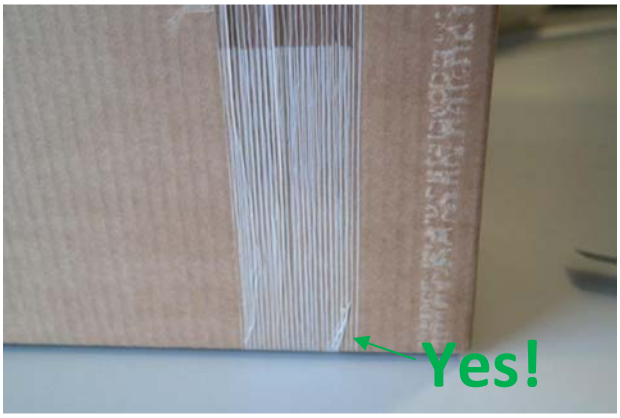

The package is now correctly packed and ready for sending, using the description in Appendix 2 of the Global Sewage Surveillance protocol.

Should you need clarification to any of the steps mentioned above, please send an email directly to suska@food.dtu.dk (Susanne Karlsmose Pedersen).

--- --- ---

Global Sewage Surveillance - SAMPLE DETAILS

Introduction

THE PURPOSE OF THIS SURVEY is to capture an overview of the collected samples.

If you have any questions or feedback for the submission of information via this survey, please contact Rene Hendriksen (rshe@food.dtu.dk), at the Technical University of Denmark.

Note: An asterisk (*) indicates a question that requires an answer.

--- --- ---

Background
No single approach exists for adequately monitoring large populations and their environments for the emergence of novel pathogens. Recent developments in high-throughput sequencing offer the ability to rapidly identify nucleic acids from various organisms in clinical and environmental samples. Sewage systems are recognized as an important source of human pathogens, especially in crowded settings with poor infrastructure.

Purpose
A point-prevalence metagenomic analysis will be applied to sewage samples collected globally from the main sewage system of major cities prior to treatment plants inlet. The project will serve as proof-of-concept for applying metagenomic approaches, which could initiate a global surveillance of human infectious diseases including antimicrobial resistance from sewage collected in major cities around the world to detect, control, prevent and predict human infectious diseases.

Procedure
From each location, two representative sewage samples (1L each, in total 2L) are collected from the main sewage flow on consecutive days from the city's main sewage pipelines prior to waste water treatment plant inlets or from the main outlet to rivers or similar. Samples can be obtained following the first filtering step, but it is important that there has been no processing of the sewage.
It is preferred to collect concentrated flow proportion sampling over 24 hours, however, should this not be possible due to lack of equipment, three crude point samples should be collected in a short time interval, i.e. at least 5 minutes between each individual sample, to ensure as much randomness as possible. Store the containers at -80°C (preferred) and prepare shipping the samples to the DTU Food in Denmark (see Appendix 2 in the protocol).
Specific description is found in the protocol sent by email from the organizer prior to sampling (for questions, please contact Rene Hendriksen, rshe@food.dtu.dk)

Global Sewage Surveillance - SAMPLE DETAILS

General Information

The following is general information relating to the contact person for the International Sewage Surveillance.

* 1. Please complete the following general information

Contact Name:

Institute name:

Address 1:

City/Town:

ZIP/Postal Code:

Country:

Email Address:

Phone Number:

* 2. SAMPLING SITE - In/near which city were the samples taken?

* 3. SAMPLING SITE - What is the name of the sampling site?

4. SAMPLING SITE - How would you characterize the sampling site? (please select one response)

- ◯ The sampling site is a waste water treatment plant
- ◯ The sampling site is an open sewer line (man-made)
- ◯ The sampling site is a river (naturally made)
- ◯ The sampling site is a stream (naturally made)
- ◯ The sampling site is a stagnant pool (naturally made)

Other (please specify)

[ ]

SAMPLING SITE - If possible, please remember to provide a digital image of the sampling site (see details described in the protocol)

* 5. SAMPLING SITE - Which geographic region does the sample cover (please describe by name of districts or areas as applicable for your locality)

[ ]

* 6. SAMPLING SITE - Is farming, slaughterhouses, industry or hospital(s) included in the area covered by the sewage sample, if so, please describe the type(s) (if not, please indicate 'no')

Farming	
Slaughterhouse(s)	
Industry	
Hospital(s)	
Other (comments)	

7. SAMPLING SITE - Which size is the area that is drained by the sampled sewage system (square km)?

[ ]

8. SAMPLING SITE - At which GPS position is the sampling site? (indicate as WGS84 geodetic datum, e.g. N43^{o}38’19.39’’ / W116^{o}14’28.86’’)

[ ]

* 9. DEMOGRAPHY - Approximately how many people live in the area covered by the sewage sample?

Approximate number of people	

* 10. DEMOGRAPHY - Which social groups live in the area covered by the sewage sample cover (please describe in general terms, e.g. upper class, middle class, lower class, slum)

[ ]

Global Sewage Surveillance - SAMPLE DETAILS

For SAMPLE NUMBER 1, please indicate the following details

* 11. For SAMPLE NUMBER 1 - Indicate assigned sample name:

[ ]

* 12. For SAMPLE NUMBER 1 - Please indicate sampling date and time (if sample is taken in a stream, indicate date and time in the first row. If sample is taken as three sub-samples, indicate date and times for each of the sub-samples (see further description in the protocol))

	DD	MM	YYYY	hh	mm	AM/PM
Sampling date and time	/	/		:		-
Sampling date and time	/	/		:		-
Sampling date and time	/	/		:		-

13. For SAMPLE NUMBER 1 - How would you characterize the flow of sewage at the sample site? (Please indicate 1, 2, 3, 4 or 5, following the graduation where 'rapid flow of sewage' is '5' and 'stagnant pool' is '1')

- ◯ 5 - At the sampling site there is a rapid flow of sewage
- ◯ 4 -
- ◯ 3 -
- ◯ 2 -
- ◯ 1 - The sample is from a stagnant pool

Other comments; also - if known - please indicate the specific velocity of the flow (m3/s)

[ ]

14. For SAMPLE NUMBER 1 - How would you characterize the viscosity of the sample? (Please indicate 1, 2, 3, 4 or 5, following the graduation where 'like water' is '5' and 'solid' is '1')

- ○ 5 - like water
- ○ 4 -
- ○ 3 -
- ○ 2 -
- ○ 1 - solid

Other (please specify)

[ ]

15. For SAMPLE NUMBER 1 - What was the temperature at the day before sampling and at the day of sampling (degrees Celcius)?

Day before sampling [ ]

Day of sampling [ ]

16. For SAMPLE NUMBER 1 - If possible, please indicate temperature of sample at sampling (degrees Celsius)

[ ]

* 17. For SAMPLE NUMBER 1 - At the day before sampling and the day of sampling, how was the weather? (please select all that apply)

- ☐ Sunny
- ☐ Cloudy
- ☐ Partly cloudy
- ☐ Foggy
- ☐ Rainy - all day rain
- ☐ Rainy - heavy showers
- ☐ Rainy - showers
- ☐ Sleeting
- ☐ Snowy
- ☐ Windy
- ☐ Stormy

Comment

[ ]

* 18. For SAMPLE NUMBER 1 - Please indicate transportation time from sample site to storage

[ ]

* 19. For SAMPLE NUMBER 1, please indicate transportation temperature from sample site to storage (degrees Celsius)

[ ]

* 20. For SAMPLE NUMBER 1, please indicate storage temperature until shipping (degrees Celsius)

[ ]

DTU

Global Sewage Surveillance - SAMPLE DETAILS

For SAMPLE NUMBER 2, please indicate the following details

* 21. For SAMPLE NUMBER 2 - Indicate assigned sample name:

[ ]

* 22. For SAMPLE NUMBER 2 - Please indicate sampling date and time (if sample is taken in a stream, indicate date and time in the first row. If sample is taken as three sub-samples, indicate date and times for each of the sub-samples (see further description in the protocol))

	DD		MM		YYYY	hh		mm	AM/PM
Sampling date and time		/		/			:		-
Sampling date and time		/		/			:		-
Sampling date and time		/		/			:		-

23. For SAMPLE NUMBER 2 - How would you characterize the flow of sewage at the sample site? (Please indicate 1, 2, 3, 4 or 5, following the graduation where 'rapid flow of sewage' is '5' and 'stagnant pool' is '1')

- ○ 5 - At the sampling site there is a rapid flow of sewage
- ○ 4 -
- ○ 3 -
- ○ 2 -
- ○ 1 - The sample is from a stagnant pool

Other comments; also - if known - please indicate the specific velocity of the flow (m3/s)

[ ]

24. For SAMPLE NUMBER 2 - How would you characterize the viscosity of the sample? (Please indicate 1, 2, 3, 4 or 5, following the graduation where 'like water' is '5' and 'solid' is '1')

- ○ 5 - like water
- ○ 4 -
- ○ 3 -
- ○ 2 -
- ○ 1 - solid

Other (please specify)

[ ]

25. For SAMPLE NUMBER 2 - What was the temperature at the day before sampling and at the day of sampling (degrees Celcius)?

Day before sampling [ ]

Day of sampling [ ]

26. For SAMPLE NUMBER 2 - If possible, please indicate temperature of sample at sampling (degrees Celsius)

[ ]

* 27. For SAMPLE NUMBER 2 - At the day before sampling and the day of sampling, how was the weather? (please select all that apply)

- [ ] Sunny
- [ ] Cloudy
- [ ] Partly cloudy
- [ ] Foggy
- [ ] Rainy - all day rain
- [ ] Rainy - heavy showers
- [ ] Rainy - showers
- [ ] Sleeting
- [ ] Snowy
- [ ] Windy
- [ ] Stormy

Comment

[ ]

* 28. For SAMPLE NUMBER 2 - Please indicate transportation time from sample site to storage

[ ]

* 29. For SAMPLE NUMBER 2, please indicate transportation temperature from sample site to storage (degrees Celsius)

[ ]

* 30. For SAMPLE NUMBER 2, please indicate storage temperature until shipping (degrees Celsius)

[ ]

Global Sewage Surveillance - SAMPLE DETAILS

ADDITIONAL INFORMATION

31. ADDITIONAL INFORMATION - At sampling, which is the season

- ◯ Spring
- ◯ Summer
- ◯ Autumn
- ◯ Winter

Comment

[ ]

32. ADDITIONAL INFORMATION - At sampling, which is the season (wet/dry)

- ◯ Wet season
- ◯ Dry season

Comment

[ ]

33. Please indicate the shipping date for the collected samples

	DD		MM		YYYY
Shipping date	[ ]	/	[ ]	/	[ ]

34. Please insert any additional comments

[ ]
