# Supplementary material for: Global monitoring of antimicrobial resistance based on metagenomics analyses of urban sewage
Source: Nat Commun. 2019 Mar 8;10:1124. doi: 10.1038/s41467-019-08853-3 (PMC6408512; doi:10.1038/s41467-019-08853-3)
Supplement: Supplementary file 2 — Peer Review File [file 41467_2019_8853_MOESM2_ESM.pdf]

Editorial Note: This manuscript has been previously reviewed at another journal that is not operating a transparent peer review scheme. This document only contains reviewer comments and rebuttal letters for versions considered at *Nature Communications*

REVIEWERS' COMMENTS:

Reviewer #2 (Remarks to the Author):

Overall the authors have addressed the comments they felt needed addressing.
